# Comparative genomics of symbiotic *Photobacterium* using highly contiguous genome assemblies from long read sequences

**DOI:** 10.1099/mgen.0.001161

**Published:** 2023-12-19

**Authors:** Alison L. Gould, James B. Henderson

**Affiliations:** ^1^​ Institute for Biodiversity Science and Sustainability, California Academy of Sciences, 55 Music Concourse Dr. San Francisco, CA 94118, California, USA

**Keywords:** bioluminescence, Oxford Nanopore Technology, symbiosis, Vibrionaceae

## Abstract

This study presents the assembly and comparative genomic analysis of luminous *Photobacterium* strains isolated from the light organs of 12 fish species using Oxford Nanopore Technologies (ONT) sequencing. The majority of assemblies achieved chromosome-level continuity, consisting of one large (>3 Mbp) and one small (~1.5 Mbp) contig, with near complete BUSCO scores along with varying plasmid sequences. Leveraging this dataset, this study significantly expanded the available genomes for *P. leiognathi* and its subspecies *P. ‘mandapamensis’*, enabling a comparative genomic analysis between the two lineages. An analysis of the large and small chromosomes unveiled distinct patterns of core and accessory genes, with a larger fraction of the core genes residing on the large chromosome, supporting the hypothesis of secondary chromosome evolution from megaplasmids in Vibrionaceae. In addition, we discovered a proposed new species, *Photobacterium acropomis* sp. nov., isolated from an acropomatid host, with an average nucleotide identify (ANI) of 93 % compared to the *P. leiognathi* and *P. ‘mandapamensis’* strains. A comparison of the *P. leiognathi* and *P. ‘mandapamensis’* lineages revealed minimal differences in gene content, yet highlighted the former’s larger genome size and potential for horizontal gene transfer. An investigation of the *lux-rib* operon, responsible for light production, indicated congruence between the presence of *luxF* and host family, challenging its role in differentiating *P. ‘mandapamensis’* from *P. leiognathi *. Further insights were derived from the identification of metabolic differences, such as the presence of the NADH:quinone oxidoreductase respiratory complex I in *P. leiognathi* as well as variations in the type II secretion system (T2S) genes between the lineages, potentially impacting protein secretion and symbiosis. In summary, this study advances our understanding of *Photobacterium* genome evolution, highlighting subtle differences between closely related lineages, specifically *P. leiognathi* and *P. ‘mandapamensis’*. These findings highlight the benefit of long read sequencing for bacterial genome assembly and pangenome analysis and provide a foundation for exploring early bacterial speciation processes of these facultative light organ symbionts.

## Data Summary

All genome assemblies are publicly available on NCBI (SAMN36714502-36714532) and the corresponding scripts used for data analysis are available on the author’s Github page (https://github.com/algould/PhotobacteriumNanopore).

**Impact Statement**

This study represents the most complete collection of *Photobacterium* genomes sequenced to date, greatly increasing the total number and quality available on NCBI. We present an effective pipeline for the assembly of highly contiguous bacterial genomes from ONT reads. By comparing these genomes, we identify key distinguishing features between closely related bacterial lineages, some of which could be important for their symbiotic interactions with a host. We also discovered a novel species of *Photobacterium* with the propsed name *P. acropomis* sp. nov.*,* isolated from the light organ of an acropomatid fish.

## Introduction

The genus *Photobacterium* belongs to the Vibrionaceae family of bacteria and contains several luminous species that form symbiotic relationships with a range of fish and squid hosts. *Photobacterium leiognathi* and its subspecies, *P. ‘mandapamensis’,* both associate with a broad range of teleost fish hosts, including fish in the Leiognathidae and Acropomatidae families as well as cardinalfish in the genus *Siphamia* (Apogonidae) [1]. *Photobacterium kishitanii *, on the other hand, is typically found in colder waters and associates with deep-dwelling fish hosts [2]. Light production in *Photobacterium* is controlled by a contiguous set of genes, termed the *lux-rib* operon. These genes can vary between species and have been used, in combination with certain housekeeping genes, to distinguish between closely related species (e.g. [ 1, 3–6]). The *luxF* gene in particular has been used as a distinguishing feature between *P. leiognathi* and other luminous *Photobacterium* species, including *P. ‘mandapamensis’; luxF* is present in most species but has been secondarily lost in *P. leiognathi* [3]. There are two additional genes located upstream of the *lux-rib* operon, *lumP* and *lumQ*, which encode proteins of the lumazine operon [7] that also vary between *P. leiognathi* and *P. ‘mandapamensis’;* like the *luxF* gene, *lumP* is present in *P. ‘mandapamensis’* but absent in *P. leiognathi *. Furthermore, two sets of orthologous genes involved in secretion can also discriminate between the two lineages [8].

Although there is notable genetic divergence between *P. leiognathi* and *P. ‘mandapamensis’*, the two groups remain indistinguishable at the 16S rRNA gene [3, 5], and the average nucleotide identity (ANI) between the two are slightly above the 95 % cut-off of the bacterial species definition [9-10], indicating the two should be considered the same species [8]. However, cardinalfish in the genus *Siphamia* appear to only associate with *P. ‘mandapamensis’* [1, 11], indicating there may be important ecological and/or physiological differences between the two groups that are recognisable by *Siphamia* hosts. For example, they differ in their growth and luminescence responses to salinity as well as the colour of light produced [3]. A previous whole genome comparison between a single strain of *P. leiognathi* and *P. ‘mandapamensis’* determined that the *P. leiognathi* strain has a larger genome with higher plasticity and a higher rate of foreign gene acquisition compared to the *P. ‘mandapamensis’* strain [8]. However, there are a limited number of genomes available with which to investigate whether these difference hold true across both lineages, the breadth of their genomic differences, and how these differences may relate to host range and specificity, particularly for the highly specific association between *P. ‘mandapamensis’* and *Siphamia* hosts.

There are currently 18 *P *. *leiognathi* genomes available from NCBI, three of which are additional assemblies of previously assembled genomes. Of the unique strains for which whole genomes are available, eight originated from the light organs of five distinct fish species, only two of which are assembled at the scaffold level. *Photobacterium leiognathi* strain *lrivu*.4.1 (GCA_000509205.1) was sequenced on the Roche 454 GS FLX Titanium platform and is comprised of 20 scaffolds [8], and the *P. ‘mandapamensis’* reference strain *svers*.1.1 (GCA_000211495.1) was sequenced on the Illumina MiSeq platform and contains 11 scaffolds [12]. The available *P *. *kishitanii* genomes are even less complete; of the 24 genomes currently available on NCBI only four are scaffolded, and only one assembly (reference strain ANT-2200) [13] contains fewer than 50 contigs. Recently, however, the genome of a *P. ‘mandapamensis’* isolate from a non-luminous *Loligo* squid was assembled using ONT sequencing [14] into only three contigs representing the large and small chromosome present in most vibrio genomes [15] as well as one plasmid sequence, showcasing the benefits of using long read sequences to assemble highly contiguous bacterial genomes.

The overall aim of this study is to characterize and compare the genome variation in symbiotic *Photobacterium* strains isolated from the light organs of diverse fish hosts in order to gain insight into the breadth of host niches occupied by these luminous symbionts as well as any genomic signatures associated with particular lineages or thier hosts. In particular, we wanted to sequence and compare the genomes of *P. leiognathi* and *P*. ‘*mandapamensis’* to gain a more complete understanding of the distinction between these two groups and to look for evidence of genomic traits and evolutionary histories associated with their respective hosts. We also include symbiotic *Photobacterium* strains isolated from several deep sea fishes to characterize their luminous symbionts and compare them to the *P. leiognathi* and *P. ‘mandapamensis’* symbionts isolated from more shallow-dwelling hosts. Leveraging ONT long read sequencing, we assembled 31 highly contiguous and near-complete *Photobacterium* genomes, including several that are fully circularized. This comprehensive genomic landscape offers insight into the genome biology of the facultative *Photobacterium* symbionts residing in fish light organs.

## Methods

### Bacterial isolates and DNA extraction

The luminous bacterial strains in this study were initially isolated from the light organs of various fish species listed in Table 1. Several strains were recently isolated from the light organs of *Siphamia tubifer* collected from Verde Island, Philippines and from Okinawa, Japan (Table 1). Those fish were handled and euthanized by a lethal dose of MS-222 in accordance with an approved protocol by the Institutional Animal Care and Use Committee at the California Academy of Sciences. The isolates were each grown on LSW-70 [1] agar plates and resuspended in liquid media overnight. Cell pellets were spun down and washed with 1 x PBS prior to DNA extraction. High molecular weight (HMW) DNA was then extracted from the fresh cell pellets using a Qiagen MagAttract HWM DNA kit following the manufacturer’s protocol. Following extraction, the DNA was purified with sparQ PureMag Beads (Quantabio) and the final DNA concentrations were determined using the Qubit dsDNA HS kit and a Qubit 3.0 fluorimeter (Thermo Fisher).

**Table 1.** Summary of the 32 *Photobacterium* sp. strains from fish light organs sequenced using Oxford Nanopore Technology. The identified species is listed as well as the host species and family from which each strain originated and the collection location and year, when available

Strain ID	Species	Host species	Host family	Location	Reference
*ahane*.1.5	* P. kishitanii *	*Acropoma hanedai*	Acropomatidae	Tungkang, Taiwan (2004)	Dunlap *et al*. [59]
*ajapo*.5.5	*P. acropomis* sp. nov.	*Acropoma japonicum*	Acropomatidae	Saga, Shikoku, Japan; Tosa Bay	Kaeding *et al*. [1]
*ajapo*.5.6	*P. ‘mandapamensis’*	*Acropoma japonicum*	Acropomatidae	Saga, Shikoku, Japan; Tosa Bay	Kaeding *et al*. [1]
*ajapo*.8.1	*P. ‘mandapamensis’*	*Acropoma japonicum*	Acropomatidae	Yui, Honshu, Japan; Suruga Bay	Kaeding *et al*. [1]
*ajapo*.8.2	*P. ‘mandapamensis’*	*Acropoma japonicum*	Acropomatidae	Yui, Honshu, Japan; Suruga Bay	Kaeding *et al*. [1]
ATCC 25521^T^	* P. leiognathi *	*Eubleekeria splendens*	Leiognathidae	Gulf of Thailand (1967)	Boisvert *et al*. (1967)
*calba*.1.1	* P. kishitanii *	*Chlorophthalmus albatrossis*	Chlorophthalmidae	Owase, Japan (2004)	Dunlap & Ast [4]
*ckamo*.1.1	* P. kishitanii *	*Coelorinchus kamoharai*	Macrouridae	Owase, Japan (2004)	Ast & Dunlap [2]
*Ik*.8.1	*P. ‘mandapamensis’*	*Siphamia tubifer*	Apogonidae	Ikei Island, Okinawa, Japan (2014)	–
*Ik*.8.2	*P. ‘mandapamensis’*	*Siphamia tubifer*	Apogonidae	Ikei Island, Okinawa, Japan (2014)	–
*Kume*.1.2	*P. ‘mandapamensis’*	*Siphamia tubifer*	Apogonidae	Kume Island, Okinawa, Japan (2014)	–
*Kume*.1.3	*P. ‘mandapamensis’*	*Siphamia tubifer*	Apogonidae	Kume Island, Okinawa, Japan (2014)	–
*lequu*.1.1	*P. ‘mandapamensis’*	*Leiognathus equula*	Leiognathidae	Manila Bay, Philippines (1982)	Dunlap *et al*. [36]; Ast & Dunlap [3]
LF-1a	*P. ‘mandapamensis’*	*Aurigequula fasciata*	Leiognathidae	Manila Bay, Philippines (1982)	Ast *et al.* [7]
*ljone*.10.1	* P. leiognathi *	*Eubleekeria jonesi*	Leiognathidae	Iloilo, Philippines (1999)	Ast *et al.* [7]
LN-1a	*P. ‘mandapamensis’*	*Nuchequula nuschalis*	Leiognathidae	Sagami Bay, Kanagawa, Japan (1980)	Ast *et al.* [7]
LN-I.1	* P. leiognathi *	*Nuchequula nuchalis*	Leiognathidae	Sagami Bay, Kanagawa, Japan (1980)	Ast *et al.* [7]
*lnuch*.19.1	*P. ‘mandapamensis’*	*Nuchequula nuchalis*	Leiognathidae	Suruga Bay, Honshu, Japan (2004)	Ast *et al.* [7]
LR-VIII.1	*P. ‘mandapamensis’*	*Equulites rivulatus*	Leiognathidae	Sagami Bay, Kanagawa, Japan (1989)	Ast *et al.* [7]
*lrivu*.20.11	*P. ‘mandapamensis’*	*Equulites rivulatus*	Leiognathidae	Suruga Bay, Honshu, Japan (2004)	Ast *et al.* [7]
*lsplen*.1.1	*P. ‘mandapamensis’*	*Eubleekeria splendens*	Leiognathidae	Gulf of Thailand (1967)	Ast *et al.* [7]
*Mot*.1.1	*P. ‘mandapamensis’*	*Siphamia tubifer*	Apogonidae	Motobu, Okinawa, Japan (2014)	–
*Mot*.1.2	*P. ‘mandapamensis’*	*Siphamia tubifer*	Apogonidae	Motobu, Okinawa, Japan (2014)	–
*pjapo*.1.1^T^	* P. kishitanii *	*Physiculus japonicus*	Moridae	Manazuru, Japan (1982)	Ast & Dunlap [3]
*StJ*.4.21	*P. ‘mandapamensis’*	*Siphamia tubifer*	Apogonidae	Motobu, Okinawa, Japan (2019)	–
*StJ*.4.81	*P. ‘mandapamensis’‘mandapamensis’*	*Siphamia tubifer*	Apogonidae	Motobu, Okinawa, Japan (2019)	–
*StP*.1.10	*P. ‘mandapamensis’*	*Siphamia tubifer*	Apogonidae	Verde Island, Philippines (2021)	–
*StP*.2.23	*P. ‘mandapamensis’*	*Siphamia tubifer*	Apogonidae	Verde Island, Philippines (2021)	–
*SV*.1.1	*P. ‘mandapamensis’*	*Siphamia tubifer*	Apogonidae	Sesoko Island, Okinawa, Japan (2008)	–
*SV*.1.2	*P. ‘mandapamensis’*	*Siphamia tubifer*	Apogonidae	Sesoko Island, Okinawa, Japan (2008)	–
*SV*.5.1	*P. ‘mandapamensis’*	*Siphamia tubifer*	Apogonidae	Sesoko Island, Okinawa, Japan (2008)	–

### Library prep and MinION sequencing

DNA concentrations were standardized across samples to an input value of 5.5 ng µl^−1^ and sequence libraries were prepared with the Rapid (96) Barcoding Kit (Oxford Nanopore Technologies) per the manufacturer’s instructions. The final libraries were pooled and sequenced on a MinION R9.4.1 flow cell. Base-calling was performed with Guppy v.6.1.7 using the ‘dna_r9.4.1_450bps_hac’ model and a quality score cutoff of eight to retain reads that were used for all subsequent analyses.

### Genome assembly

After base-calling, the sequence reads were additionally filtered with Filtlong v0.2.1 (https://github.com/rrwick/Filtlong), removing reads less than 1000 bp and applying various ‘keep_percent’ settings (80, 90, and 95 %). Draft genome assemblies were produced from these sets of filtered reads using the Flye v2.9 assembler [16]. Circlator v1.5.5 [17] was then run on the draft assemblies to attempt to circularize any additional contigs, followed by two polishing steps. The first round of polishing was carried out with Medaka v1.6.0 (https://github.com/nanoporetech/medaka) followed by Homopolish v0.3.4 [18] with ‘* Photobacterium *’ provided as the input genus. After polishing, additional genome scaffolding was carried out using both RagTag v2.1.0 [19] and Ragout v2.3 [20]. The highest quality, circularized and polished draft assemblies produced by Flye were used as references for scaffolding along with the reference strain JS01 (GCA_002631085.2). For the *P. kishitanii* strains, the reference genome (ANT-2200, GCA_002631085.1), which has the fewest number of contigs (*n*=5) of the *P. kishitanii* genomes available from NCBI, was used for scaffolding.

Two strains, *StP*.2.23 and *StJ*.4.81, also had Illumina short reads (150 bp paired-end reads) available from a recent study [21] that were used along with the ONT reads as input for Unicycler v0.5.0 [22] to produce hybrid assemblies. The short reads were first quality filtered and trimmed using fastp v0.23.2 [23]. The resulting assemblies were then circularized and scaffolded with Circlator v1.5.5 [17] and RagTag v2.1.0 [19], respectively, and compared with their long read-only assemblies. All programmes were implemented in separate Conda v4.14.0 environments.

### Annotation and genome comparisons

BUSCO v5.3.2 [24] scores were calculated using the Vibrionales (vibrionales_odb10) set of genes (*n*=1445) throughout the assembly pipeline to assess completeness. Similarly, Prokka v1.14.6 [25] was implemented to annotate the draft assemblies at each step and to compare gene content and number. QUAST v5.0.2 [26] was also used to calculate genome statistics at various steps. Whole genome comparisons were made between all pairwise combinations of strains using FastANI v1.33 [27], and ANIclustermap v1.2.0 [28] was implemented to visualize the results. Additionally, a synteny analysis was carried out between one of the most complete *P.* ‘mandapamensis’ assemblies, strain *Ik*.8.2, and the reference strain *svers*.1.1 (GCA_000211495.1) with Sibelia v3.0.7. All programmes were run in separate Conda v4.14.0 environments.

### Plasmids and mobile genetic elements

Plasmids were identified with plasmidVerify v20Apr2022 [29] on the complete genome assemblies. Additional contigs smaller than chromosome one and two that were not identified by plasmidVerify but were fully circularized during the assembly were also considered to be a plasmid. Prokka v1.14.6 [25] was implemented in Conda v4.14.0 on these additional contigs to obtain the plasmid gene content. Mobile genetic elements (MGEs) were also identified by running MobileElementFinder v1.1.2 [30] on the assembled genomes.

### Pangenome and phylogenetic analysis

A pangenome analysis of the *P. leiognathi* and *P. ‘mandapamensis’* strains was carried out with Roary v3.13.0 [31] based on the Prokka annotations of the final assemblies. Additional reference strains available from NCBI (Table 2) were also annotated with Prokka and included in the analysis for comparison. The pangenome results were then used to identify genes that were distinct to either the *P. leiognathi* or *P. ‘mandpamensis’* genomes using the query_pan_genome function in Roary. Chromosome-specific Prokka annotations were also used as Roary input to produce alignments of the core genes present on each chromosome. The core genome alignments were then used to construct maximum likelihood phylogenies in IQ-TREE v2.0.3 [32] using the best predicted model and a maximum of 1000 bootstrap replicates. Additional phylogenetic analyses were carried out on two orthologs previously identified by Urbanczyk et al. [8] that differentiate *P. leiognathi* from *P. ‘mandapamensis’* as well as on a set of genes identified in Roary as unique between the two groups. All programmes were implemented in Conda v4.14.0.

**Table 2.** *
Photobacterium* genomes available from NCBI included in the study

Genbank ID	Strain ID	Species
GCA_003026895.1	A2-4	* Photobacterium angustum *
GCA_009665375.1	2012 V-1072	* Photobacterium damselae *
GCA_002954725.1	JCM 21184	* Photobacterium phosphoreum *
GCA_000613045.3	ANT-2200	* Photobacterium kishitanii *
GCA_000509205.1	*lrivu*.4.1	* Photobacterium leiognathi *
GCA_003026025.1	*ajapo*.4.1	*Photobacterium ‘mandapamensis’*
GCA_003026055.1	*Res*.4.1	*Photobacterium ‘mandapamensis’*
GCA_003026695.1	AJ-1a	*Photobacterium ‘mandapamensis’*
GCA_003026735.1	*ajapo*.3.1	*Photobacterium ‘mandapamensis’*
GCA_000211495.1	*svers*.1.1	*Photobacterium ‘mandapamensis’*
GCA_002631085.2	JS01	*Photobacterium ‘mandapamensis’*

## Results

### MinION sequencing data

The ONT MinION sequencing run generated 5.29M fast5 reads with an N50 of 9.1 Kb. After demultiplexing and base calling with Guppy, a total of 1.76M reads (4.82 Gbp) were obtained, 145 744 of which were unclassified (no barcode could be assigned). The number of reads assigned to each sample ranged from 7816 to 122 045, and the minimum and maximum sequencing depths were 4× and 130×, respectively.

### Draft genome assemblies

The initial Flye assemblies on the filtered long reads (90 ‘keep percent’) were first visualized with Bandage [33] (Fig S1 available in the online version of this article). The genome of one strain, *ajapo*.5.5, was assembled as two complete circular chromosomes, one large (>3 100 000 bp) and one small (>1 400 000 bp), with one additional circular plasmid (~16 000 bp). Nine additional assemblies contained at least one circular chromosome (Fig. S1). After running Circlator on the Flye assemblies, two additional chromosomes were circularized from two different assemblies, and the total number of contigs decreased for nearly all of the assemblies (Fig. S2). In contrast, the polishing steps had no effect on the number of contigs, but did increase the BUSCO completeness scores, in some cases substantially. For example, strains LR-VIII.1 and *StP*.2.23 went from completeness scores of 42 and 41% to 77 and 80 %, respectively. Running Homopolish after initial polishing with medaka especially improved BUSCO scores across all strains, and in some cases, there was a greater than 20 % increase in completeness (Fig. S3). Scaffolding, on the other hand, had little to no effect on the BUSCO scores, but did decrease the number of contigs even further for nearly all assemblies. After scaffolding, 16 of the 32 strains ended up with draft genomes that were comprised of only two or three contigs (Table S1). The most notable scaffolding improvement was observed for strain *StJ*.4.81, which went from 50 to three contigs. Scaffolding also increased the number of coding sequences (CDS) detected for most strains (Table S1). In the case of *StJ*.4.81, the total number of CDS increased from 4346 to 4361, while the number of rRNAs and tRNAs remained the same, 63 and 208, respectively. One strain, *StJ*.4.33, had low average coverage (3.4 ×) and the BUSCO completeness score only reached 2.8 %. Thus, it was removed from further analysis. Of the remaining 31 strains, 27 had BUSCO completeness scores of 95 % or greater, 15 of which were 99 % complete (Fig. S3).

**Fig. 1.**
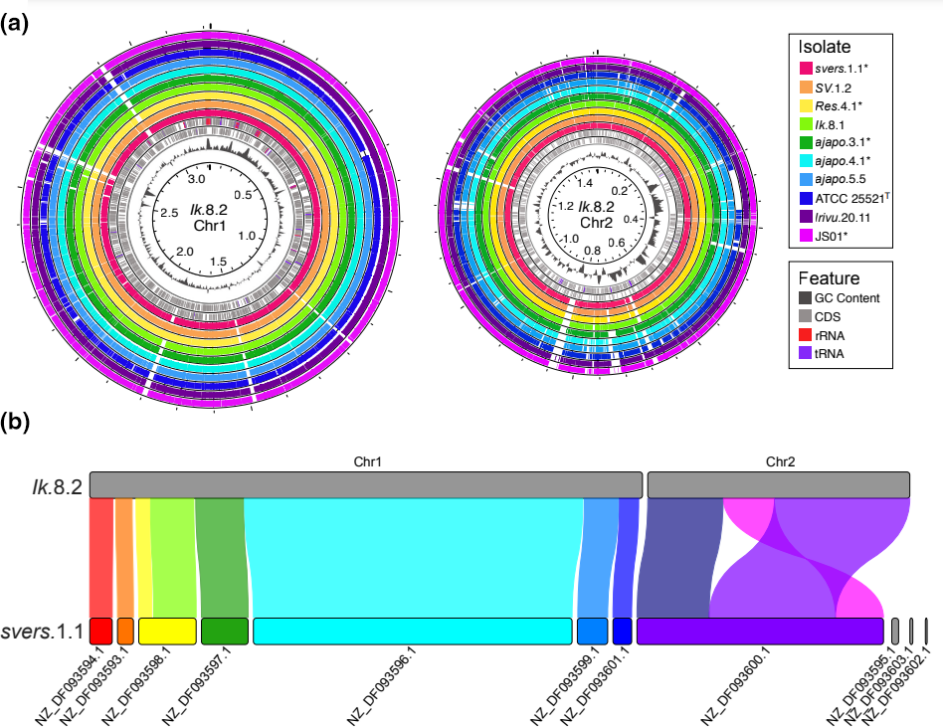
Synteny of the genome assembly of isolate *Ik.8.2* to that of other *P. leiognathi* and *P*. ‘*mandapamensis’* genomes. (**a**) blastn alignments of the isolates listed to both chromosomes of *Ik.8.2*. Assemblies from NCBI are indicated with an *. Genomic features including coding sequences (CDS), rRNAs, tRNAs, and GC content are shown on the inner rings of each chromosome. (**b**) Syntenic blocks between the *P*. ‘*mandapamensis’* reference strain *svers*.1.1 (GCA_000211495.1) and the chromosome-level assembly of *Ik*.8.2. Figure was produced with RIdeogram [60].

For the final assemblies, the average genome size was 4 944 424 bp across all 32 strains, ranging from 4 521 083 to 5 791 416 bp, including four *P. kishitanii* strains. The *Photobacterium leiognathi* and *P. ‘mandapamensis’* strains averaged 4 920 253 total bp and had an average of 4393 CDS, 49 rRNAs, and 193 tRNAs. The *P. leiognathi* strains were approximately 6 % larger than the *P. ‘mandapamensis’* genomes, whereas the four *P. kishitanii* strains were even larger, averaging 5 085 414 total bp with an average of 5576 CDSs, 33 rRNAs, and 196 tRNAs (Table 3). The average size of each chromosome also varied between the *P. kishitanii, P. leiognathi* and *P. ‘mandapamensis’* strains. The average size of the large chromosome (chr1) was 3.32 Mbp, 3.36 Mbp, and 3.27 for the *P. kishitanii, P. leiognathi*, and *P. ‘mandapamensis’* strains, respectively. The average size of the small chromosomes (chr2) were 1.64 Mbp for *P. kishitanii* strains, 1.53 Mbp for the *P. leiognathi* strains, and 1.53 Mbp for the *P. ‘mandapamensis’* strains. The assembly for strain *Ik*.8.2, which was isolated from a *Siphamia tubifer* light organ from Okinawa, Japan in 2014, consisted of three circular contigs, representing both the large and small chromosome and a plasmid. There were 2741 CDSs, 181 tRNAs, and 56 rRNAs on the large chromosome (chr1) and 1320 CDSs, 27 tRNAs, and no rRNAs on the small chromosome (chr2). Similarly, strain *ajapo*.5.5, which had the highest depth of coverage of all strains, contained 2696 CDSs, 181 tRNAs, and 59 rRNAs on the large chromosome and 1286 CDSs, 27 tRNAs, and no rRNAs on the small chromosome. A blast (34) comparison of *P. ‘mandapamensis’* strain *Ik*.8.2 to several other strains revealed multiple unique gene regions that were only found in *Ik*.8.2 (Fig. 1), although most of the genes in these regions were of unknown function. A comparison of the genome assembly of strain *Ik*.8.2 and the reference strain *svers*.1.1, indicates a high degree of genome synteny and exemplifies the ability of this highly contiguous assembly to be used to scaffold previous assemblies available from NCBI (Fig. 1b).

**Table 3.** Statistics for the final genome assemblies as determined by QUAST. Reference strains indicated with an * are also included for comparison. Listed are the number of total contigs, the number of contigs greater than 1000, 10 000, and 50 000 bp, the largest contig in bp, the total number of bp, %GC content, the N50 and L50 values, and the number of N’s per 100 Kbp. The first five shaded entries are *Photobacterium kishitanii* strains whereas all others are *P. leiognathi* and *P. ‘mandapamensis’* strains.

Strain	Total bp	Contigs	Largest contig	N50	L50	GC(%)	**ns**	CDS	rRNA	tRNA
*ahane*.1.5	5 100 844	5	3 353 657	3 353 657	1	39.02	126	4680	53	207
*calba*.1.1	5 200 973	7	3 263 220	3 263 220	1	38.96	792	4892	46	206
*ckamo*.1.1	5 039 339	4	3 317 370	3 317 370	1	38.96	2489	6136	8	173
*pjapo*.1.1^T^	5 000 498	4	3 339 696	3 339 696	1	39.36	12 224	6596	25	196
*pjapo*.1.1^T^*	4 695 065	117	925 439	174 214	6	39.10	0	4217	6	84
AJ-1a*	4 711 244	65	468 721	238 774	7	41.14	0	4156	13	131
*ajapo*.3.1*	4 794 394	51	480 625	245 626	8	40.98	0	4214	12	137
*ajapo*.4.1*	4 576 643	52	696 744	262 428	5	41.2	0	3991	11	124
*ajapo*.5.5	4 690 822	3	3 177 212	3 177 212	1	41.37	0	4004	59	307
*ajapo*.5.6	4 729 792	2	3 174 233	3 174 233	1	41.27	0	4052	62	213
*ajapo*.8.1	4 878 649	4	3 245 602	3 245 602	1	41.12	0	4255	44	192
*ajapo*.8.2	4 886 267	3	3 261 968	3 261 968	1	41.15	0	4239	53	194
ATCC 25521^T^	4 750 881	3	3 269 131	3 269 131	1	41.02	0	4255	41	194
*Ik*.8.1	4 765 281	3	3 231 342	3 231 342	1	41.15	0	4130	47	197
*Ik*.8.2	4 783 140	3	3 218 875	3 218 875	1	41.17	0	4104	56	207
JS01*	4 874 529	3	3 251 164	3 251 164	1	41.20	0	4288	57	205
*Kume*.1.2	4 835 311	3	3 186 767	3 186 767	1	41.25	1634	4156	49	197
*Kume*.1.3	4 818 955	3	3 185 044	3 185 044	1	41.23	1911	4137	43	194
*lequu*.1.1	4 825 624	4	3 204 041	3 204 041	1	41.19	0	4192	62	209
LF-1a	5 104 659	5	3 267 821	3 267 821	1	40.96	1222	4607	39	192
*ljone*.10.1	5 276 714	8	3 313 411	3 313 411	1	41.34	1	4535	62	207
LN-1a	5 174 290	10	3 359 026	3 359 026	1	41.21	54	4760	44	195
LN-I.1	5 490 629	15	3 405 483	3 405 483	1	41.53	20	5017	62	200
*lnuch*.19.1	5 237 683	5	3 439 577	3 439 577	1	41.18	162	4618	57	197
LR-VIII.1	5 791 416	21	3 699 906	3 699 906	1	41.28	22 860	5602	32	147
*lrivu*.20.11	4 738 701	2	3 228 638	3 228 638	1	41.24	0	4069	58	209
*lrivu*.4.1*	5 268 214	20	1 730 671	979 827	2	40.98	6943	4332	3	72
*lsplen*.1.1	5 292 468	7	3 284 001	3 284 001	1	41.01	813	4768	37	174
*Mot*.1.1	4 676 757	2	3 973 505	3 973 505	1	41.25	13	4069	62	208
*Mot*.1.2	4 947 312	2	3 234 211	3 234 211	1	41.2	2558	4336	62	206
*Res*.4.1*	4 730 847	65	766 940	129 576	9	40.99	0	4098	13	152
*StJ*.4.21	4 877 430	4	3 228 436	3 228 436	1	41.23	623	4361	63	208
*StJ*.4.81	4 713 802	3	3 157 280	3 157 280	1	41.22	6	4047	45	195
*StP*.1.10	4 781 050	3	3 175 608	3 175 608	1	41.15	19	4194	47	194
*StP*.2.23	4 685 648	3	3 130 376	3 130 376	1	41.17	11	4043	33	182
*SV*.1.1	4 749 844	2	3 174 417	3 174 417	1	41.13	1321	4126	38	190
*SV*.1.2	4 718 353	2	3 184 478	3 184 478	1	41.17	0	4083	51	200
*SV*.5.1	4 521 083	2	3 089 627	3 089 627	1	41.27	91	4122	35	193
*svers*.1.1*	4 598 918	11	1 910 320	1 477 894	2	41.06	742	4031	6	75

### Hybrid assemblies

The use of short reads improved the assembly for the two strains for which they were available, *StP*.2.23 and *StJ*.4.81. After trimming, there were 3 385 214 and 5 808 314 paired-end 150 bp reads for *StP*.2.23 and *StJ*.4.81, respectively that were used for the initial assembly step in Unicycler v0.5.0 [22]. With respect to BUSCO scores, the hybrid assemblies were more complete than the long read-only assemblies. For strain *StP*.2.23, the BUSCO completeness score went from 77.8 to 99.1 % for the Flye and Unicycler assemblies, respectively, and for *StJ*.4.81, it improved from 94.5 to 99.1 % (Table S3). Running both Circlator [17] and RagTag [19] on the assemblies reduced the number of contigs but had slightly negative effects on the BUSCO scores. The hybrid assembly for *StP*.2.23 went from 30 contigs down to two scaffolds, but the BUSCO completeness score decreased to 96.3 %. Similarly, the hybrid assembly for strain *StJ*.4.81 went from 15 to two contigs after both circularizing and scaffolding, but BUSCO completeness dropped to 98.6 % (Table S3). However, removing sequences of fewer than 1000 bp from the scaffolded (non-circularized) hybrid assemblies resulted in only three contigs for both strains and 99.1 % BUSCO completeness scores, and were used in the remaining analyses for strains *StP*.2.23 and *StJ*.4.81.

### Average Nucleotide Identity

**Fig. 2.**
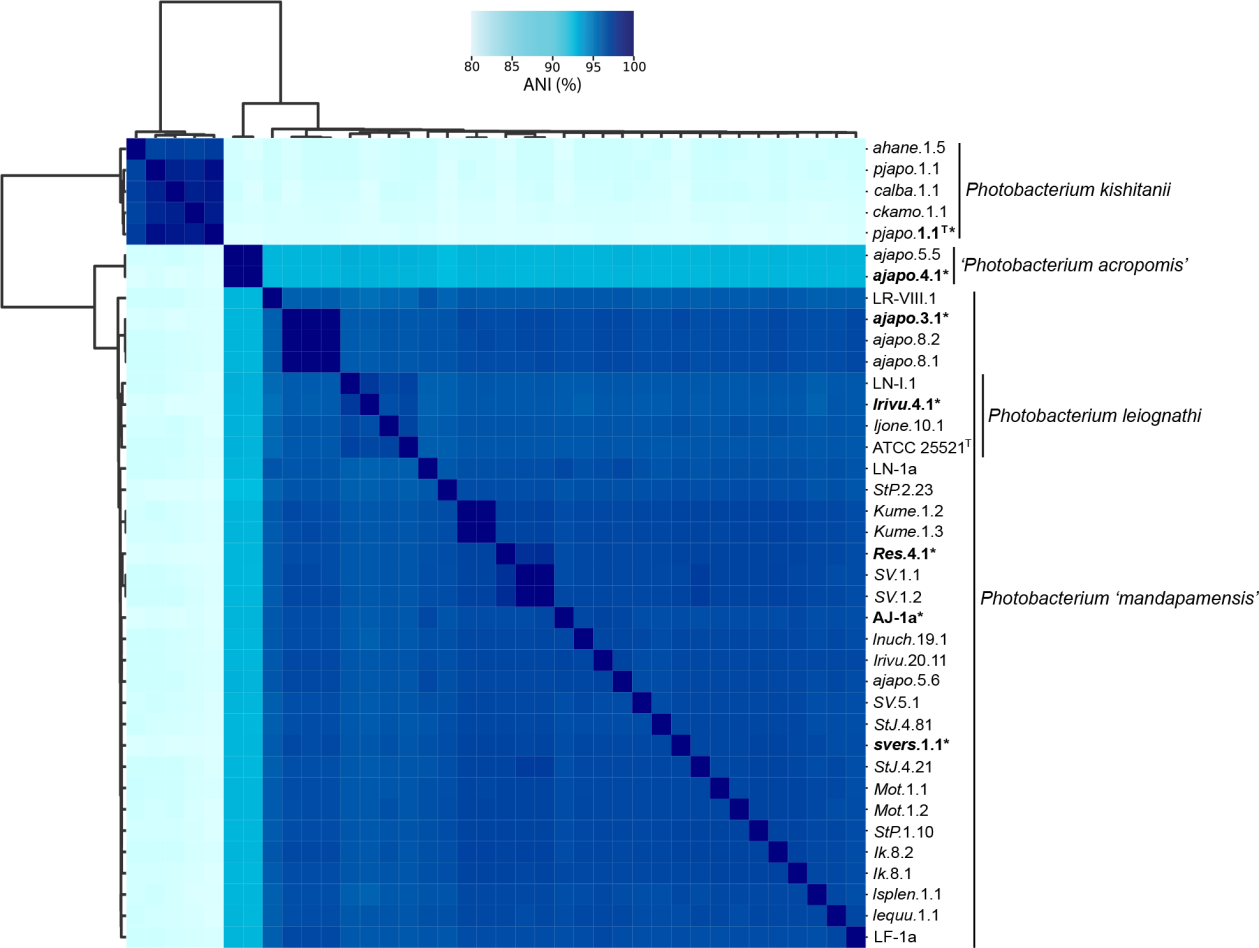
Clustered heatmap depicting the pairwise average nucleotide identities (ANI) of the *Photobacterium* strains sequenced in this study. Reference strains included in the analysis are in bold and indicated with an *. Corresponding ANI values are listed in Table S2.

The pairwise ANI analysis across all strains showed a clear distinction between the *P. kishitanii* strains and all of the others, with an average ANI of 80.6 % between the two groups (Fig. 2, Table S2). The pairwise comparisons among all non-* P. kishitanii* strains resulted in ANIs greater than 95 %, with the exception of two strains, *ajapo*.5.5 and *ajapo*.4.1. The average ANI values between these two strains and the *P. leiognathi* and *P. ‘mandapamensis’* strains is 93.03 and 92.86 %, respectively, both below the 95 % threshold suggested for bacterial species delimitation [9-10](Fig. 2). Thus, we propose these two strains are representatives of a new species, *Photobacterium acropomis* sp. nov., and strain *ajapo5.5* has been deposited in the SeqCode registry as such [35]. The average pairwise ANI between the *P. leiognathi* strains and *P. ‘mandapamensis’* strains was 96.5 %, whereas the *P. leiognathi and P. ‘mandapamensis’* strains were 97.3% and 97.1% similar to themselves, respectively.

### Plasmids and mobile genetic elements

Of the plasmid sequences identified across all strains, 24 were fully circularized. The average length of these circularized sequences ranged from 7304 to 100 280 bp with a mean of 39 194 bp. Most strains had only one identifiable plasmid, although three circularized plasmids were identified in strains *lsplen*.1.1 and *LN*-I.1. Four additional strains contained two plasmids. The majority of genes identified across all plasmid sequences were of unknown function, but there were 163 total genes that were assigned function, 30 of which encoded transposases, and 52 that were shared by at least two strains (Fig. S4). The remaining 111 genes were uniquely found in only a single strain (Table S4). Comparing genes across the plasmid sequences from different strains revealed some similarities between plasmids originating from the same host species and location, such as *ajapo*.8.1/*ajapo*.8.2 and *Ik*.8.1/*Ik*.8.2. There were also several genes present in plasmids across all strains, including *bin3*, *dns*, *repA*, and *tnpR* (Fig. S4). With respect to transposases, the IS6 family transposase ISPpr9 was the most common across strains.

An analysis of MGEs indicated that 19 of the sequenced strains contain no MGEs, and the remaining strains had between one and 252 MGEs (Table S5). The two strains with the highest number of MGEs (LN-I.1 and *ljone*.10.1) were both *P. leiognathi* strains, and the top seven strains with the most MGEs were isolated from leiognathid hosts (Fig. S5, Table S5). In contrast, only four of the strains isolated from apogonid hosts (all *P. ‘mandapamensis’)* contained a single MGE. Both *P. acropomis* sp. nov. strains (*ajapo*.4.1 and *ajapo*.5.5) lacked MGEs as well, and only one *P. kishitanii* strain (*calba*.1.1) contained three MGEs (Table S5). Of the MGEs identified, most were classified as insertion sequences ranging in size from 785 bp to 2593 bp. All others were identified as composite transposons with highly variable lengths, ranging from 1308 bp to 51 845 bp (Fig. S6).

### *Lux-rib* operon

**Fig. 3.**
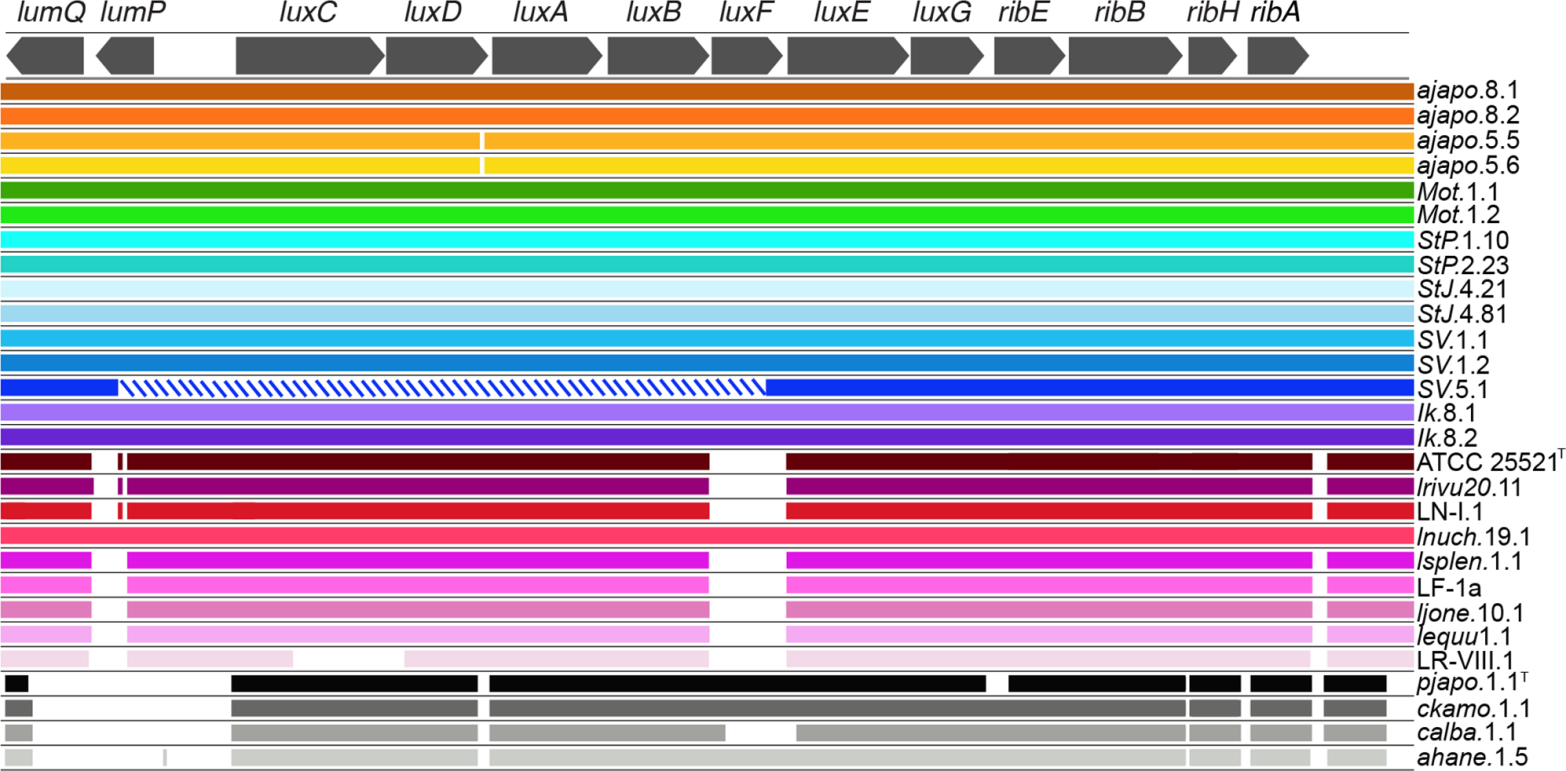
Alignment of the *lux-rib* operon of the *Photobacterium sp*. isolates sequenced in this study. *Photobacterium ‘mandapamensis’* strain *svers*.1.1 (12) was used as the reference for blast comparisons of the other isolates using an e-value cutoff score of 0.1. The isolate names are listed to the right of their respective colour band. Hash marks indicate an incomplete genome assembly. Figure produced with Proksee [61].

A comparison of the *lux-rib* operon of the different strains revealed a pattern that corresponds with the host family from which the bacteria originated. Bacteria isolated from *Acropoma japonicum* (‘*ajapo*’ strains) and *Siphamia tubifer* hosts all contain the *luxF* gene (Fig. 3). One strain, *SV*.5.1, which was isolated from the light organ of *S. tubifer,* had an incomplete assembly of the *lux* genes and is thus, excluded from this analysis. In contrast, all strains that were isolated from the light organs of leiognathid fishes, with the exception of one strain, *lnuch*.19.1, did not contain *luxF* as well as most of the *lumP* gene (Fig. 3). The four *P. kishitanii* isolates all contained *luxF* but lacked both *lumP* and *lumQ*.

### Pangenome analysis

**Fig. 4.**
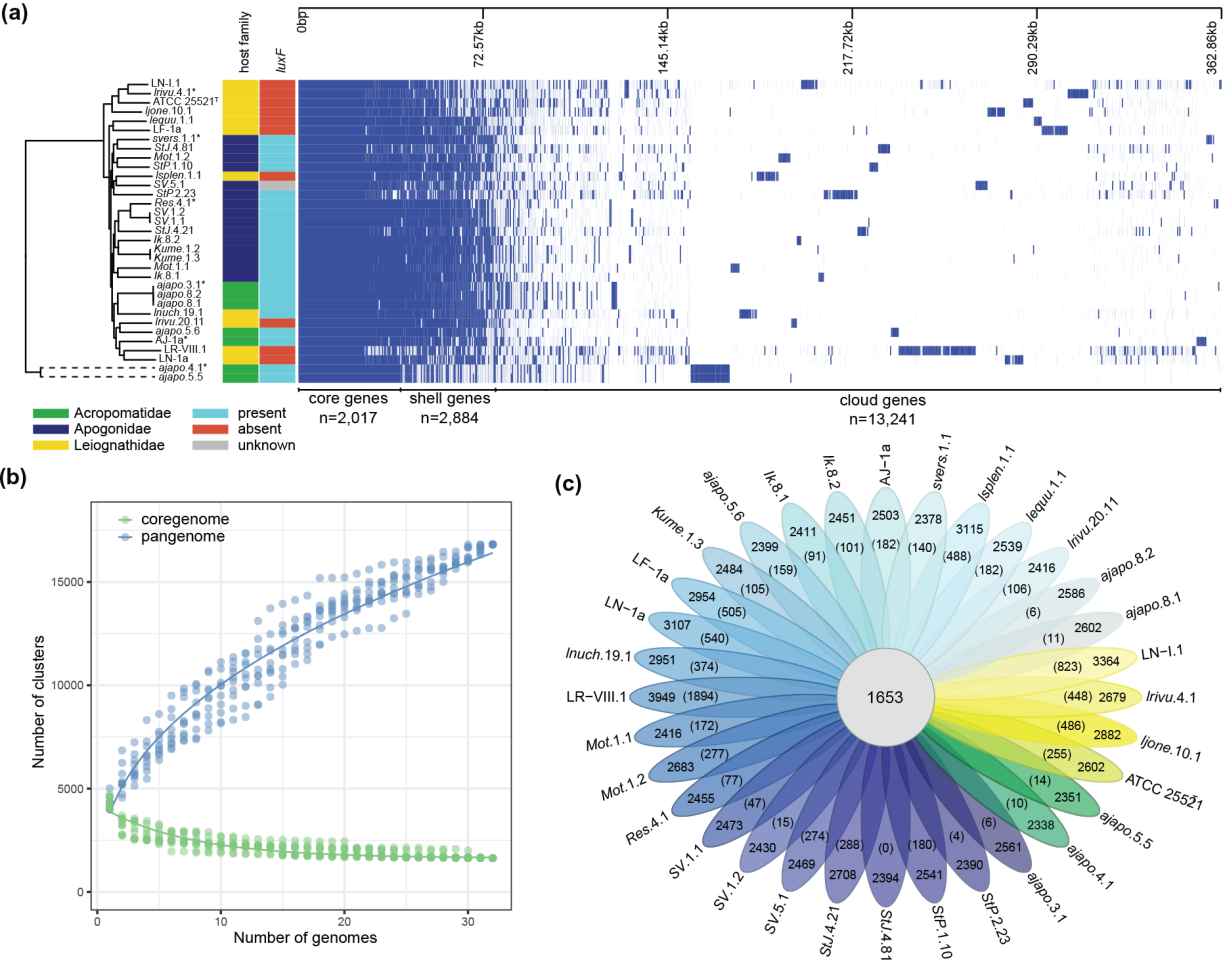
Pangenome analysis of *Photobacterium leiognathi* and *P. ‘mandapamensis’* strains isolated from the light organs of various fish hosts. (a) Phandango [62] plot of gene presence and absence across the core genome the strains where blue indicates the presence of a gene and white indicates its absence. A phylogenetic tree of the strains is also shown as well as each strain’s corresponding host family of origin and the presence or absence of *luxF* in the genome as indicated by the corresponding colours to the right of the phylogeny (see legend for details). (b) Plot of the number of gene clusters in the core- (green) and pan- (blue) genomes as a function of the number of genomes examined. (c) Flower plot showing the number of shared and unique genes between the strains. The number in the middle indicates the number of shared genes across all strains. The numbers in parentheses indicate the number of unique genes identified for each strain.

A pangenome analysis revealed a total of 18 142 genes across all *P. leiognathi* and *P. ‘mandapamensis’* strains examined in this study. Of these, 2017 genes are ‘core’ genes shared across at least 95 % of the strains and 2884 are ‘shell’ genes shared across 15–95 % of strains. The majority of the genes detected (73 %, *n*=13 241) are ‘cloud’ genes present in fewer than 15 % of the total strains examined, and of these, 9664 were singletons, present in only a single genome (Fig. 4). A separate analysis of the *P. ‘mandapamensis’* strains (*n*=27) revealed a pangenome of 2618 genes. The two *P. acropomis* sp. nov. strains (*ajapo*.5.5 and *ajapo*.4.1), are divergent from the *P. leiognathi* and *P. ‘mandapamensis’* strains and share 787 genes that are not present in any of the other strains examined, 136 of which are of unknown function (Fig. 4). Of the remaining genes with assigned function, several are related to vitamin B12 transport (*btuCDF*), the synthesis of enterobacterial common antigen (*wecABC*), and urease production (*ureABCDEFG*) (Table S6).

**Fig. 5.**
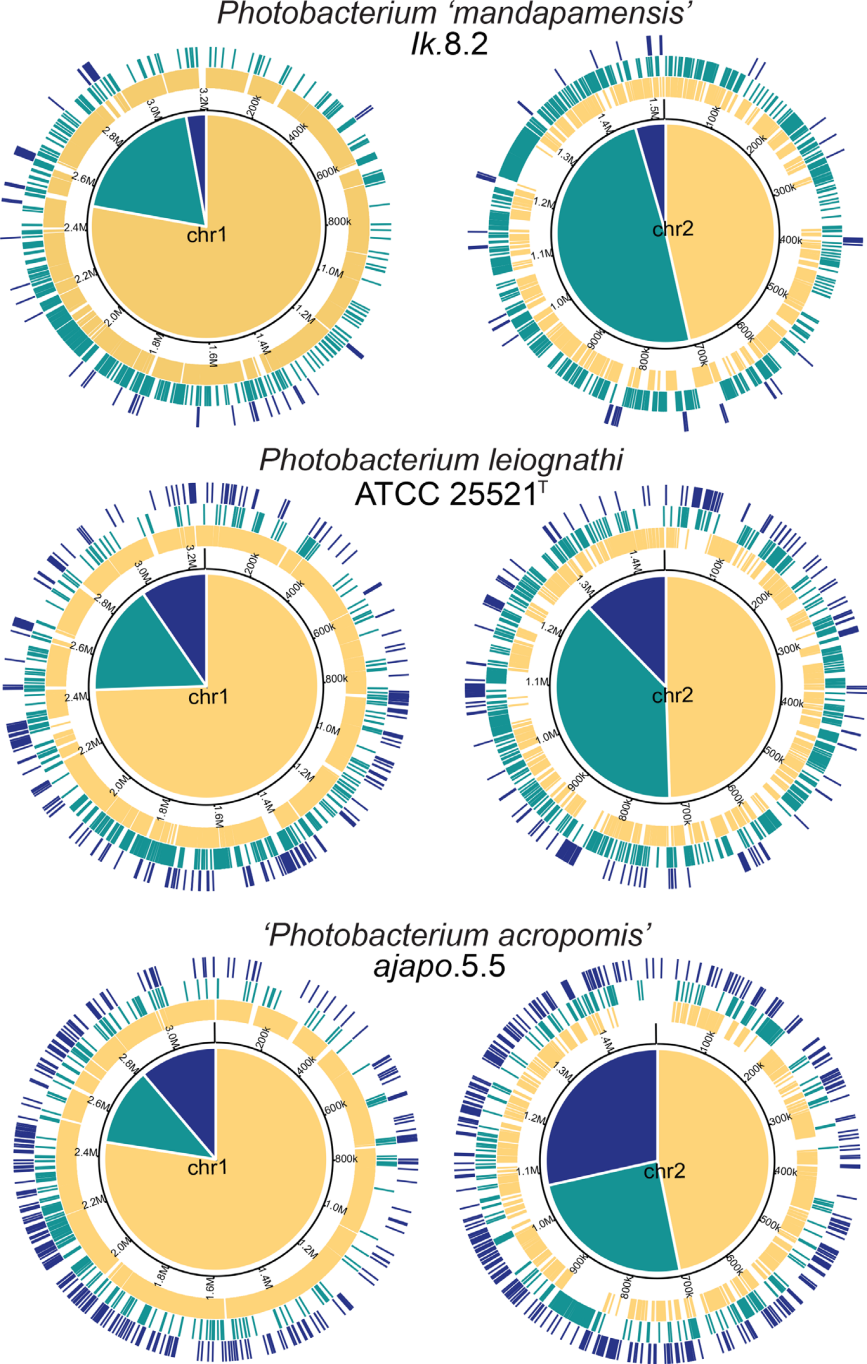
Uneven distribution of the core (yellow), shell (green), and cloud (blue) genes on the large (chr1) and small (chr2) chromosomes for representative genomes of three *Photobacterium* isolates sequenced in this study. Pie charts in the centre of each plot depict the relative proportion of bps of each gene type present on that chromosome.

An analysis of the core, shell, and cloud genes on each chromosome indicated that they are unevenly distributed between the two chromosomes (Fig. 5). For *P. ‘mandapamensis’* strain *Ik*.8.2, chromosome one contains more core genes relative to its size than on chromosome two. In contrast, chromosome one contains a lower number of shell genes than chromosome two, despite being over twice as large. The ratio of core:shell genes on chromosome one was approximately 4.5 : 1 versus closer to 1 : 1 on chromosome two. This uneven distribution of core and shell genes between chromosome one and two was also true for *P. leiognathi* strain ATCC 25521^T^ as well as for *Photobacterium acropomis*. sp. nov. strain *ajapo*.5.5 (Fig. 5).

### Phylogenetic analysis

**Fig. 6.**
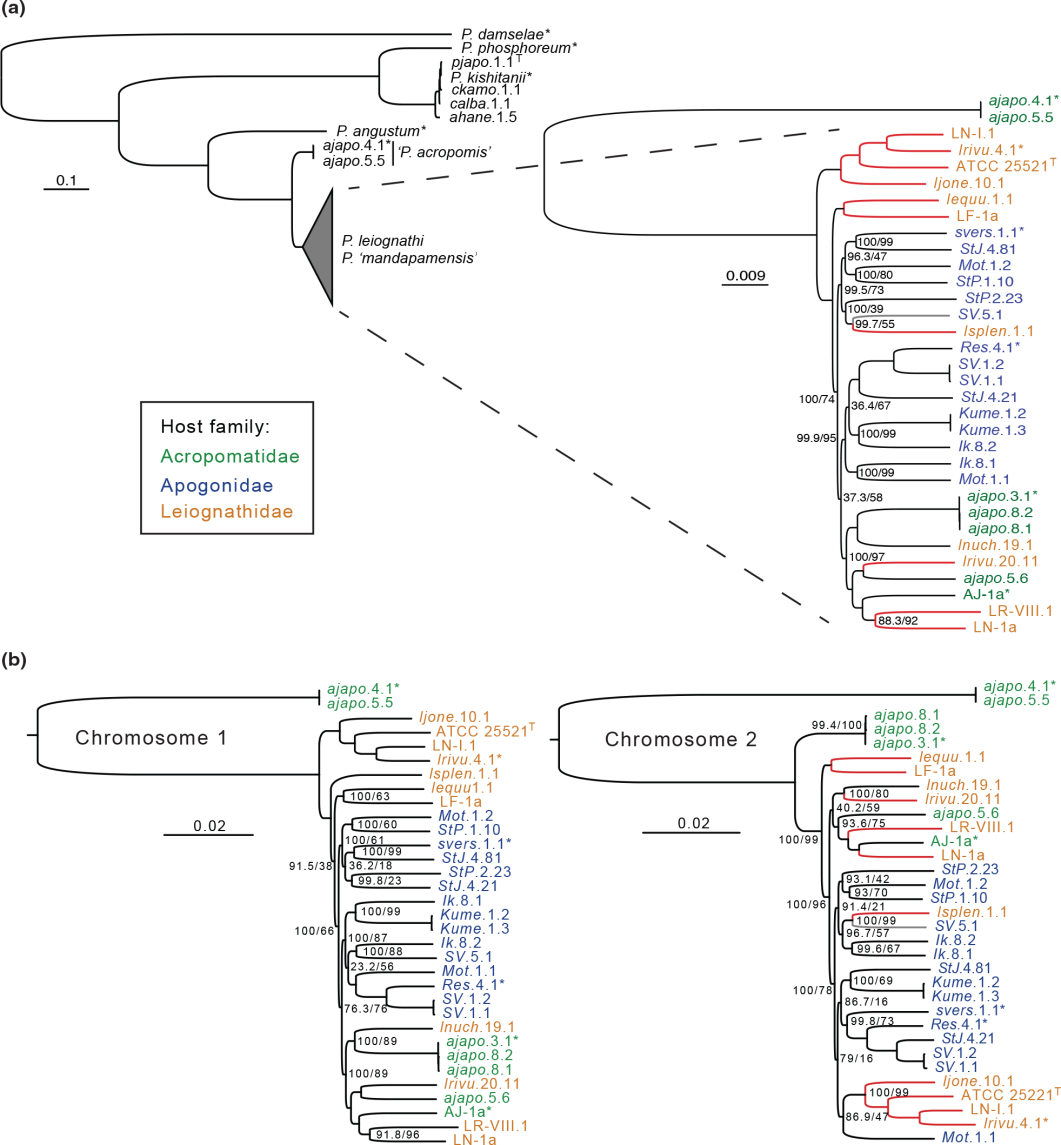
Phylogenetic analysis of *Photobacterium* species isolated from the light organs of various fish hosts. (**a**) Midpoint rooted trees are shown for an analysis of all strains sequenced in this study based on an alignment of 520 core genes with 150 bootstrap replicates and only the *P. leiognathi* and *P*. ‘*mandapamensis’* strains based on an alignment of 2017 core genes with 300 bootstrap replicates. (**b**) Inferred phylogenies of the *P. leiognathi* and *P. ‘mandapamensis’* strains based on an alignment of the core genes present on chromosome one (*n*=1636) and chromosome two (*n*=229) with 145 and 200 bootstrap replicates, respectively. Tip labels are coloured according to which host fish family the strain originated from. Red branches indicate the absence of *luxF,* which is located on chromosome two. Reference strains from NCBI are indicated by an *. Scale bars show the inferred number of nucleotide substitutions per site. All trees were constructed using the GTR+F+I+G4 model in IQ-TREE. Node values indicate bootstrap/SH-aLRT support. Nodes missing values indicate 100/100 support.

A phylogenetic analysis based on an alignment of 520 core genes identified across all strains, including the four *P. kishitanii* strains and NCBI reference strains representative of additional *Photobacterium* species, indicates strong support for *P. leiognathi/’mandapamensis’* clade (Fig. 6a). This analysis also supports the divergence of strains *ajapo*.4.1 and *ajapo*.5.1 from this clade. An additional analysis of these two strains and the *P. leiognathi* and *P. ‘mandapamensis’* strains based on an alignment of 2017 core genes detected in the pangenome analysis indicated four distinct clades among the *P. leiognathi* and *P. ‘mandapamensis’* strains in this study. Two of the clades are comprised entirely of strains originating from the host fish, *Siphamia tubifer*, with the exception of a single strain, *lsplen*.1.1, which originated from the Leiognathid host, *Eubleekeria splendens*. A third clade is comprised of strains that originated from the light organs of both Leiognathid and Acropomatid fishes, and a fourth clade, basal to the other three, contain only strains originating from Leiognathid hosts, including the *P. leiognathi* type strain ATCC 25521^T^ (Fig. 6a). This clade also contains *P. leiognathi* strain *lrivu*.4.1 (GCA_000509205.1), whereas the other three clades contain strains previously identified as *P. ‘mandapamensis’*. This analysis also placed strains *ajapo*.5.5 and *ajapo*.4.1 (GCA_003026025.1) as divergent from the *P. leiognathi* and *P. ‘mandapamensis’* strains with high confidence (100/100) (Fig. 6b). The inferred phylogeny based on the two orthologous genes previously identified by Urbanczyk *et al*. [8] as distinct between *P. leiognathi* and *P. ‘mandapamensis’* support the overall tree topology from the core gene alignment with the exception of the placement of the candidate species *P. acropomis* sp. nov.*,* which is nested between the *P. leiognathi* and *P. ‘mandapamensis’* strains, but remains a distinct clade (Fig. S5).

The separate phylogenetic analyses of the core gene alignments for chromosome one and two highlight differences in their evolutionary histories (Fig. 6b). The topology of the chromosome one tree is nearly identical to that of the tree based on the entire set of core genes and places the *P. leiognathi* strains together as sister to the *P. ‘mandapamensis’* strains. The placement of one strain, *lsplen*.1.1*,* as sister to all other *P. ‘mandapamensis’* strains in the chromosome one tree is different from both the complete core gene tree and the chromosome two tree. Additionally, there is little congruence between the chromosome one and two trees. In the chromosome two tree, the *P. leiognathi* clade is nested among the *P. ‘mandapamensis’* strains and is sister to strain *Mot*.1.1, although with relatively low support (87/47). Furthermore, a group of strains originating from Acropomatid hosts (*ajapo*.8.1, *ajapo*.8.2, *ajapo*.3.1*) is sister to the remaining *P. leiognathi* and *P. ‘mandapamensis’* strains. Furthermore, *luxF* appears to have been lost multiple times, as the absence of this gene is not limited to one clade in either the chromosome two tree or the complete core gene tree. However, the loss of *luxF* does appear to be a synapomorphy of *P. leiognathi* (Fig. 6).

### Differences between *P. leiognathi* and *P. ‘mandapamensis’*

A comparison of the unique genes between the *P. leiognathi* and *P. ‘mandapamensis’* strains with Roary revealed 40 genes present in all *P. ‘mandapamensis’* strains that were absent in the four *P. leiognathi* strains (Table S7) and 24 genes that were present in all *P. leiognathi* strains but absent in all *P. ‘mandapamensis’* strains (Table S8). Among the genes unique to *P. leiognathi* were several genes in the *nuo* operon, which encodes the energy-converting NADH:quinone oxidoreductase respiratory complex I. Additionally, six genes involved in type II secretion (*gspC, gspD, gspJ, gspK, gspL, and gspM*) were reciprocally identified as distinct between the two sets of strains by Roary and were confirmed to be divergent from one another in a phylogenetic analysis (Fig S7).

## Discussion

Using ONT sequencing, we were able to assemble highly contiguous genomes of 31 *Photobacterium* species originating from the light organs of 12 species of fish representing six unique families in four orders. The majority of the assemblies were at the chromosome-level, comprised of one contig greater than 3 Mbp and a smaller contig approximately 1.5 Mbp. Several strains had additional plasmid sequences ranging in size from approximately 2000–100 000 bp. These values are consistent with what has been reported for the genomes of other *Photobacterium* species, which are typically comprised of one large and small chromosome between 3.13 to 4.09 Mbp and 1.05 to 2.24 Mbp, respectively, as well as numerous small plasmids [6, 15, 36-37]. The study represents the more complete collection of *Photobacterium* genomes sequenced to date, enabling a more robust analysis of the closely related lineages *P. leiognathi* and *P. ‘mandapamensis’*. This set of highly contiguous genomes also allowed us to compare average nucleotide identities across strains and identify a proposed new species of *Photobacterium, P. acropomis* sp. nov.*,* isolated from the light organ of an acropomatid fish.

There are many programmes currently available for the assembly of bacterial genomes from ONT sequences, and previous studies have compared some of the different assembly approaches (e.g. [38, 39, 40, 41], yet no clear consensus pipeline has been developed. Comparing several combinations of various assembly tools used in this study, we chose a pipeline that implemented the Flye assembler [16] followed by Circlator [17] to orient and circularize the sequences as well as both Medaka and Homopolish [18] for polishing. After polishing we ran Ragout [20] or RagTag [19] for scaffolding with varying results depending on the strain. Overall, this pipeline resulted in the assembly of highly contiguous, near-complete *Photobacterium* genomes, even from samples with less than 10× sequence coverage depth. Similar to other studies [39, 41], we found that using Homopolish as a secondary polishing step dramatically improved BUSCO completeness scores, particularly for strains with poor coverage depth. We also saw a reduction in the number of contigs for many of the assemblies after running Circlator and, most notably, after scaffolding. Not surprisingly, incorporating available short reads into the assembly with the hybrid assembler Unicycler [33] also greatly increased BUSCO completeness scores. The two hybrid assemblies did contain a larger number of contigs than did the long read-only assemblies, primarily due to a large number of short contigs, but these numbers were reduced significantly by scaffolding and discarding short contigs (<1000 bp).

Due to the highly contiguous nature of the *Photobacterium* genomes assembled, we were also able to examine differences in the gene content and the evolutionary histories of both the large and small chromosomes. There was an uneven distribution of the core genes between the two chromosomes, with three-quarters of chromosome one being made up of core genes as opposed to only half of chromosome two. In contrast, a much larger fraction of chromosome two was made up of shell and cloud genes. This pattern has been observed in other *Vibrionaceae* genomes [42] and suggests that the large chromosome is more evolutionarily constrained, which explains the discrepancies in the inferred phylogenies between the chromosomes. This difference is seen in other bacterial genomes composed of multiple chromosomes, such as *Burkholderia* or *Vibrio* species, where the larger chromosome tends to contain more conserved housekeeping genes and have greater content preservation and overall synteny [43-44]. A leading hypothesis explaining the origin of secondary chromosomes in the Vibrionaceae is that they evolved from megaplasmids [43], thus explaining the greater genetic variability of these smaller chromosomes, which can serve as accessory genomes for certain conditions or environmental niches. Furthermore, the content of secondary chromosomes appears to be predisposed to evolve more rapidly [44]. Our examination of gene content and the evolutionary histories of the large and small chromosomes across *Photobacterium* strains revealed asymmetrical distribution patterns of core and accessory genes, supporting the hypothesis of secondary chromosome evolution from megaplasmids.

The *lux-rib* operon, responsible for light production, is located on the smaller chromosome and has rapidly diverged within the Vibrionaceae [45]. Our results indicate that the presence of *luxF* does not correlate with the phylogenetic relationships of the strains, but rather there is strong congruence between the presence of *luxF* and host family of origin. In fact, several strains inferred to be *P. ‘mandapamensis’* based on their phylogenetic positioning are lacking *luxF*, suggesting its presence or absence is not a defining feature between *P. leiognathi* and *P. ‘mandapamensis’* as previously thought [3]. All strains originating from Apogonidae (genus *Siphamia*) and Acropomatidae (genus *Acropoma*) hosts in this study contained *luxF*, whereas all strains from Leiognathid hosts were lacking this gene, with the exception of one strain that was most closely related to strains isolated from acropomatid hosts. This could indicate that a major selective pressure acting on this operon is the host niche. While *luxF* is not required for light production, its presence appears to increase light emission [21, 46], which may be required for the bioluminescent symbiosis with *Siphamia* and *Acropoma* hosts, and perhaps, is a key feature for these hosts to recognize specific symbionts from the environmental pool of bacteria.

Despite the congruence of host range and the presence of the *luxF* gene, there remains no evidence of the bioluminescent symbiosis having played a role in the divergence of *P. leiognathi* and *P. ‘mandapamensis’*. Furthermore, strains from both lineages have been identified as co-symbionts of the same light organ [1]. While the four *P. leiognathi* strains in this study all originated from leiognathid hosts, the host range of the *P. ‘mandapamensis*’ strains was much broader, with hosts from the Leiognathidae, Acropomatidae, and Apogonidae families, although a larger number of strains were sequenced from this lineage. With respect to the host’s symbiont range, leiognathid fishes can associate with a wide range of both *P. leiognathi* and *P. ‘mandapamensis’* strains. Acrompomatids may have an even broader range, as we discovered that they can also associate with the proposed new species, *P. acropomis* sp. nov. In contrast, apogonid hosts in the genus *Siphamia* associate with a much narrower range of *P. ‘mandapamensis’* strains [1, 11, 21]. It remains unclear why this degree of specificity exists for the bioluminescent symbiosis with *Siphamia* and not for the other hosts examined, but it could be due to the fish’s distinct behavioural ecology as a cryptic reef fish [47]. Furthermore, there are likely mechanisms in place for the *Siphamia* hosts to identify *P. ‘mandapamensis’,* such as the presence of *luxF* and other key genomic features.

This study confirms that *P. leiognathi* and *P. ‘mandapamensis’* are phylogenetically and ecologically closely related and are at the ANI cut-off typically used to delimit bacterial species [9, 10], providing a unique opportunity to analyse the early stages of bacterial speciation [8]. With these high-quality genome assemblies, we were able to compare the pangenomes of these two lineages to gain a better understanding of their defining differences. The *P. leiognathi* genomes in this study are, on average, 6 % larger than the *P. ‘mandapamensis’* strains, and their core genome is also larger*,* although this could be a result of the smaller number of *P. leiognathi* strains that were sequenced. A previous study suggested that *P. leiognathi* has a more plastic genome and acquires genes horizontally more frequently than *P. ‘mandapamensis’* [8]. Aligned with those results, the *P. leiognathi* genomes in this study contained a larger number of MGEs, which can facilitate horizontal gene transfer [48]. Additionally, the *P. ‘mandapamensis’* strains with the largest number of MGEs were isolated from leiognathid hosts, whereas those isolated from apogonid hosts contained few to none, which could indicate key differences in the host light organ environments making them more amenable to MGEs. Alternatively, this could indicate selectivity by the hosts for particular strain types. Nevertheless, the *Photobacterium* pangenome overall does appear to be larger than that of other members of the *Vibrionaceae* [49], which may be the reason for their ability to colonize different environmental niches [50]. Horizontal gene transfer has been documented from more distantly related bacteria to *Photobacterium* species, contributing to the large pangenome size [6]. The role of plasmids in the genome evolution of *Photobacterium* has not been thoroughly examined, however, our study confirms previous findings that *Photobacterium* plasmids typically lack essential genes [51]. Nevertheless, more comprehensive investigations of the plasmids in these strains using more traditional methods, such plasmid isolation and sequencing, would provide better insight into their role in these facultative light organ symbionts.

Overall, the differences in gene content between the *P. leiognathi* and *P. ‘mandapamensis’* genomes were minimal, and the two lineages have an average ANI of 96.5 %, however there were some unique genes identified between the two groups, such as the presence of the *nuo* operon in the *P. leiognathi* strains. The *nuo* genes encode the NADH:quinone oxidoreductase respiratory complex I, a homolog of the mitochondrial respiratory enzyme of the same name, which often acts as the main respiratory NADH dehydrogenase in bacteria [52]. In many marine species, the electron transport system possesses a redox-driven sodium pump (Nqr) instead of the NADH:ubiquinone oxidoreductase (Nuo) [53]. The Nqr-NADH dehydrogenase complex couples electron transport to Na^+^ translocation across the membrane [54] and thus, enables marine bacteria to maintain viable internal conditions in highly saline environments [53]. The *P. leiognathi* strains examined contained both energy-conserving NADH dehydrogenases, whereas the *P. ‘mandapamensis’* (and *P. kishitanii *) strains only contained the Nqr-NADH dehydrogenase complex. Studies of other bacterial strains containing both operons have shown that they can compensate for one another and provide metabolic flexibility in oxidizing NADH under a variety of conditions [55].

Another key difference between the *P. leiognathi* and *P. ‘mandapamensis’* strains in this study was evident in their type II secretion (T2S) systems. T2S is one means by which symbiotic gram-negative bacteria secrete proteins into the host environment [56] and has been linked to the export of virulence factors in many pathogenic bacteria [57]. It may also be involved in transporting proteins that have a role in mutualistic associations between bacteria and eukaryotic hosts [56]. In fact, in another bioluminescent symbiosis, the secreted halovibrins HvnA and HvnB, may have a role in the persistence of *Vibrio fischeri* symbionts in their squid hosts [58]. While all of the *Photobacterium* strains sequenced contain all 12 T2S genes, six of those genes (*gspC, gspD, gspJ, gspK, gspL, and gspM*) are distinct between the *P. leiognathi* and *P. ‘mandapamensis’* strains. Although it is unknown if these differences have any effect on protein secretion between the two groups, these findings warrant an investigation into the role of TS2 in the bioluminescent symbiosis of these bacteria.

In conclusion, this study utilized ONT sequencing to assemble highly contiguous genomes of 31 *Photobacterium* strains isolated from the light organs of diverse fish hosts. The resulting dataset is the most complete collection of *Photobacterium* genomes to date and enabled the identification of a proposed new species, *P. acropomis* sp. nov. The described assembly pipeline proved effective in generating near-complete genomes even from low-coverage samples. The analysis of these genomes uncovered significant differences in gene content between the large and small chromosomes, reinforcing the evolutionary constraints on the former. Additionally, this study highlights the congruency of the presence of *luxF* in the *lux-rib* operon with host family rather than the evolutionary history of the strains, challenging previous assumptions about the role of this gene in distinguishing *P. ‘mandapamensis’* from *P. leiognathi *. A pangenome comparison did reveal subtle differences between these lineages, such as the presence of the respiratory complex I in *P. leiognathi* and divergence in their T2S genes, suggesting potential metabolic and symbiotic adaptations. Overall, this study enhances our understanding of the genomic evolution of these facultatively symbiotic *Photobacterium* species, particularly among the closely related *P. leiognathi* and *P*. ‘*mandapamensis’* lineages*,* setting the stage for further investigations into the early stages of bacterial speciation and the potential role of host associations in this process.

## Supplementary Data

Supplementary material 1
