# Supplementary material for: Comparative genomics of symbiotic Photobacterium using highly contiguous genome assemblies from long read sequences
Source: Microb Genom. 2023 Dec 19;9(12):001161. doi: 10.1099/mgen.0.001161 (PMC10763503; doi:10.1099/mgen.0.001161)
Supplement: Supplementary material 1 [file mgen-9-1161-s001.pdf]

Summary

This document contains supplemental figure and tables for the manuscript: “Highly contiguous genome assemblies and a new species of *Photobacterium* isolated from fish light organs” by AL Gould and JB Henderson.

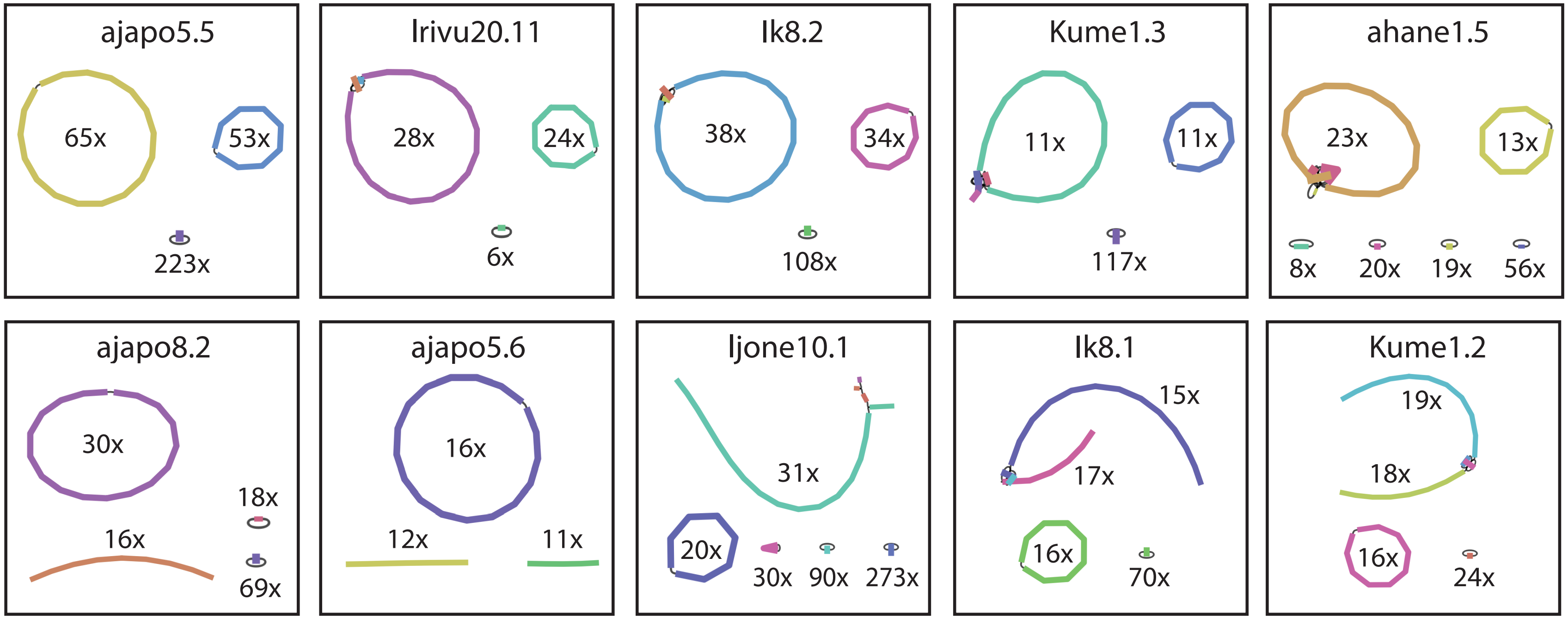

Figure S1. Bandage assembly plots for the ten *Photobacterium* strains with at least one fully circularized chromosome from the initial Flye assembly. Coverage depth of each contig is also indicated. Images created with Bandage (Wick *et al.* 2015).

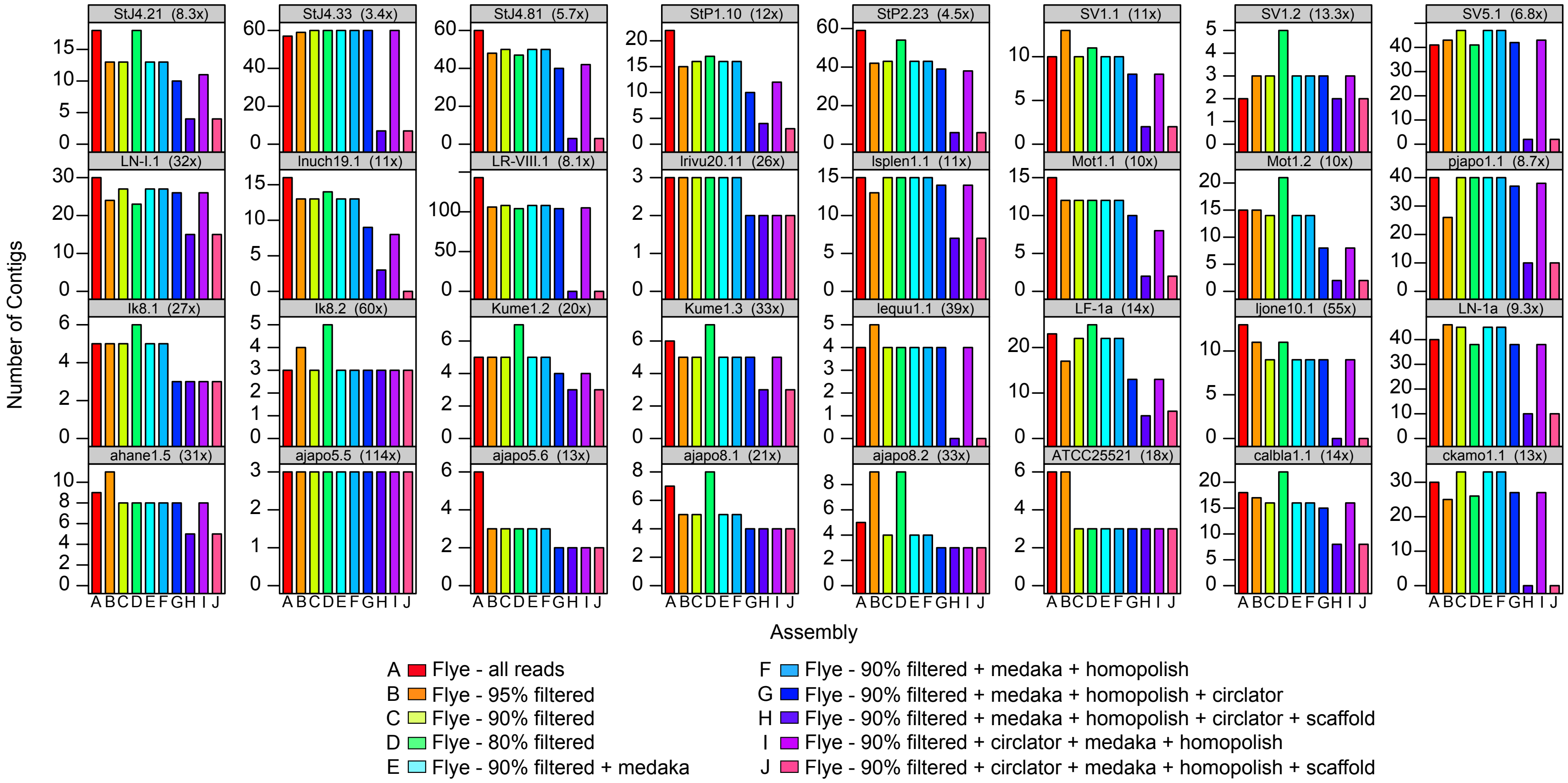

Figure S2. Number of contigs for each draft assembly of the 32 strains of *Photobacterium* using only ONT reads. The strain names and their average coverage depth are indicated in the gray bar above each plot. The different assembly approaches are indicated in the legend and colored accordingly.

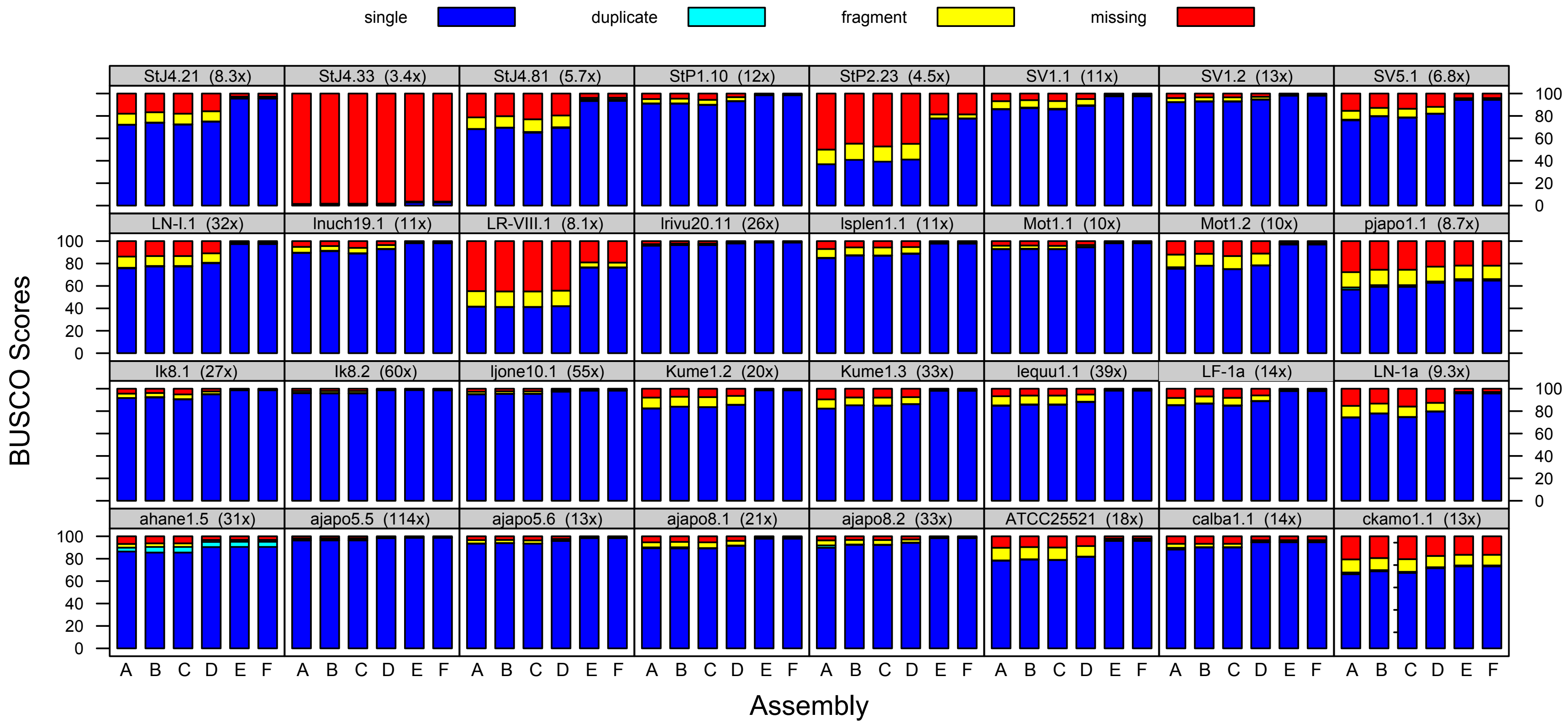

A: Flye - 80% filtered
B: Flye - 90% filtered
C: Flye - 90% filtered + circlator
D: Flye - 90% filtered + circlator + medaka
E: Flye - 90% filtered + circlator + medaka + homopolish
F: Flye - 90% filtered + circlator + medaka + homopolish + scaffold

Figure S3. BUSCO scores for the draft assemblies of the 32 strains of *Photobacterium* using only ONT reads. The strain names and their average coverage depth are indicated in the gray bar above each plot. The different assembly approaches are indicated in the legend and the bar colors represent the different BUSCO gene categories: single copy (blue), duplicate (cyan), fragmented (yellow), and missing (red).

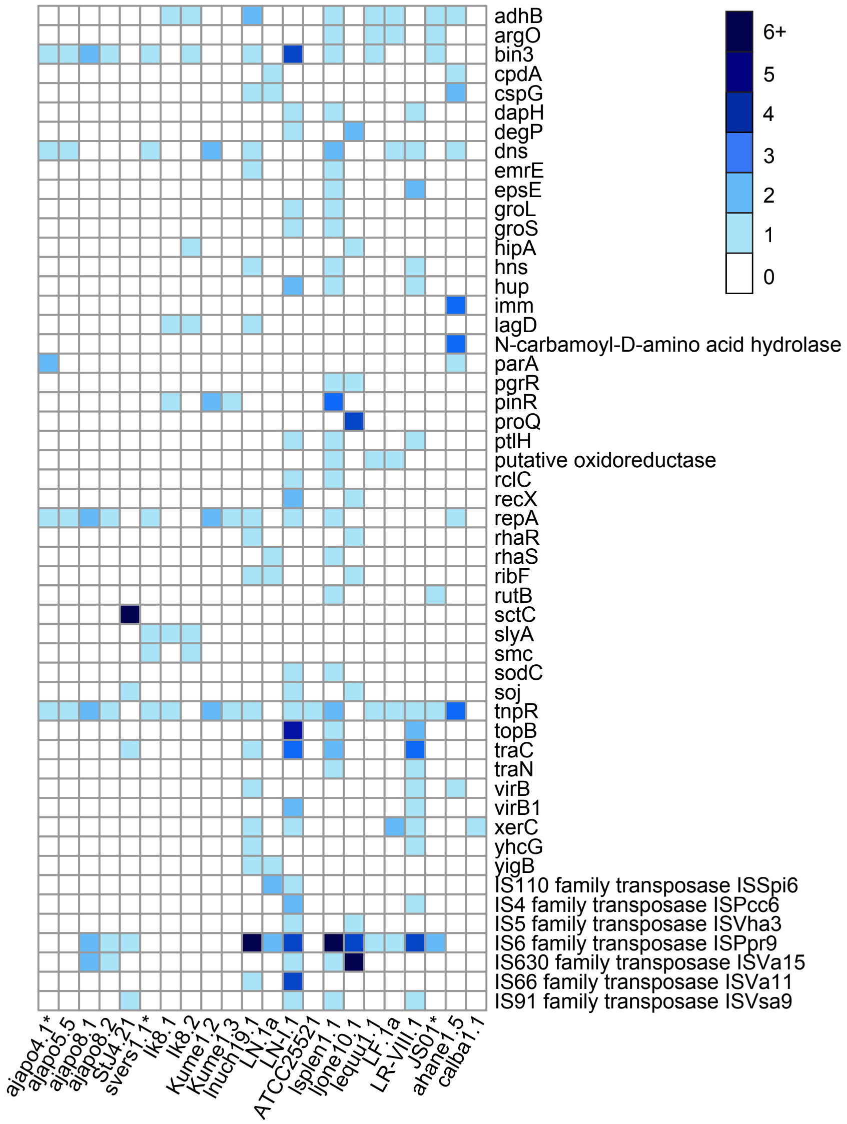

Figure S4. Summary of plasmid gene content of the *Photobacterium* strains sequenced in this study. Genes listed are present in at least two strains and their copy number is indicated by the corresponding legend color. A complete list of genes present in all plasmid sequences identified are presented in Table S3. Reference strains included in the analysis are indicated with a *.

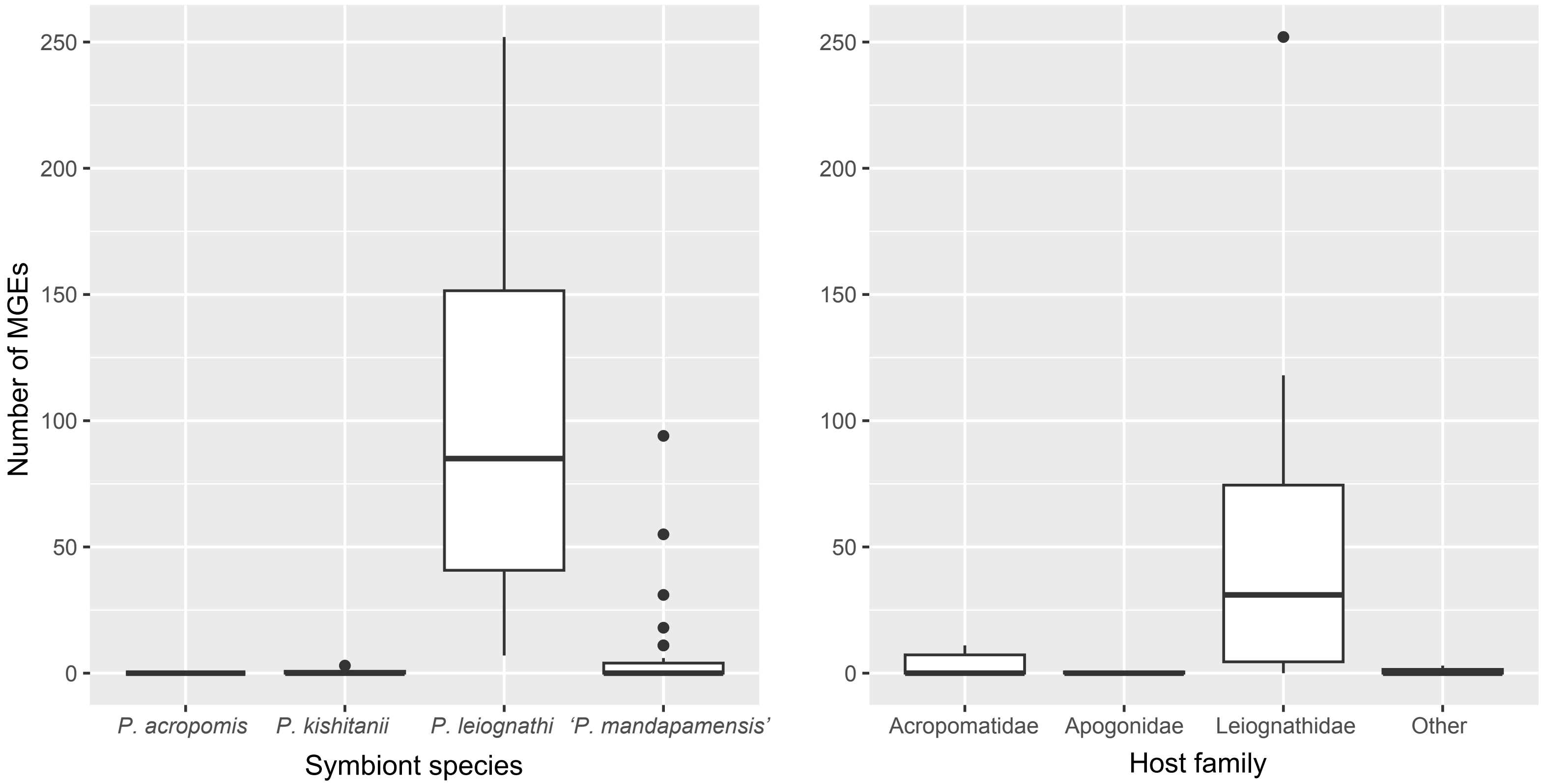

Figure S5. The number of mobile genetic elements (MGE) detected in each bacterial symbiont genome. Symbionts are grouped by bacterial species (left) or from which host fish family the strain originated (right).

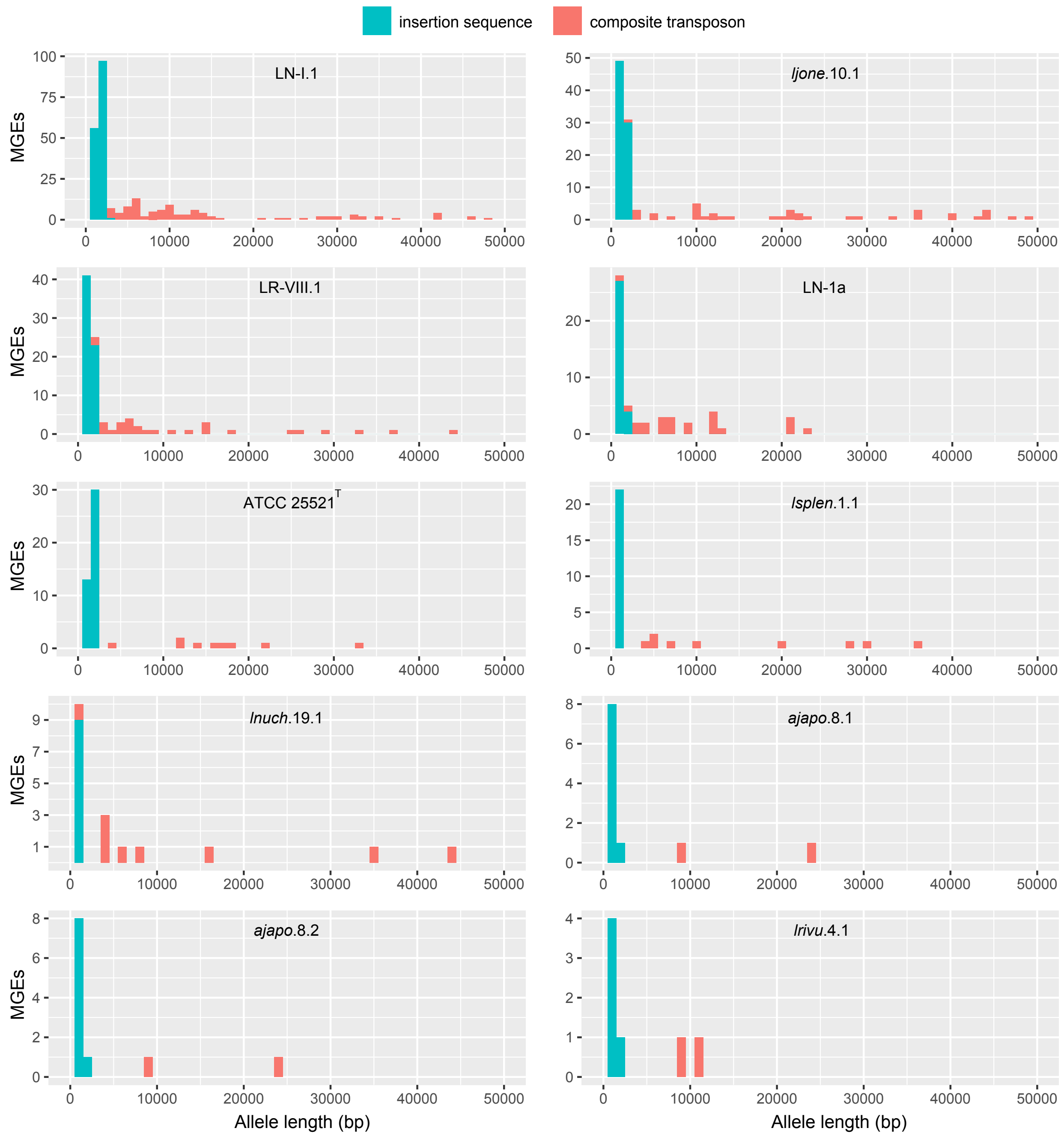

Figure S6. Size profiles of the mobile genetic elements (MGE) identified in the ten *Photobacterium* genomes with the highest number of MGEs. The MGE type (insertion sequence or composite transposon) are designated by the color indicated in the legend above. The symbiont strain is indicated at the top of each plot.

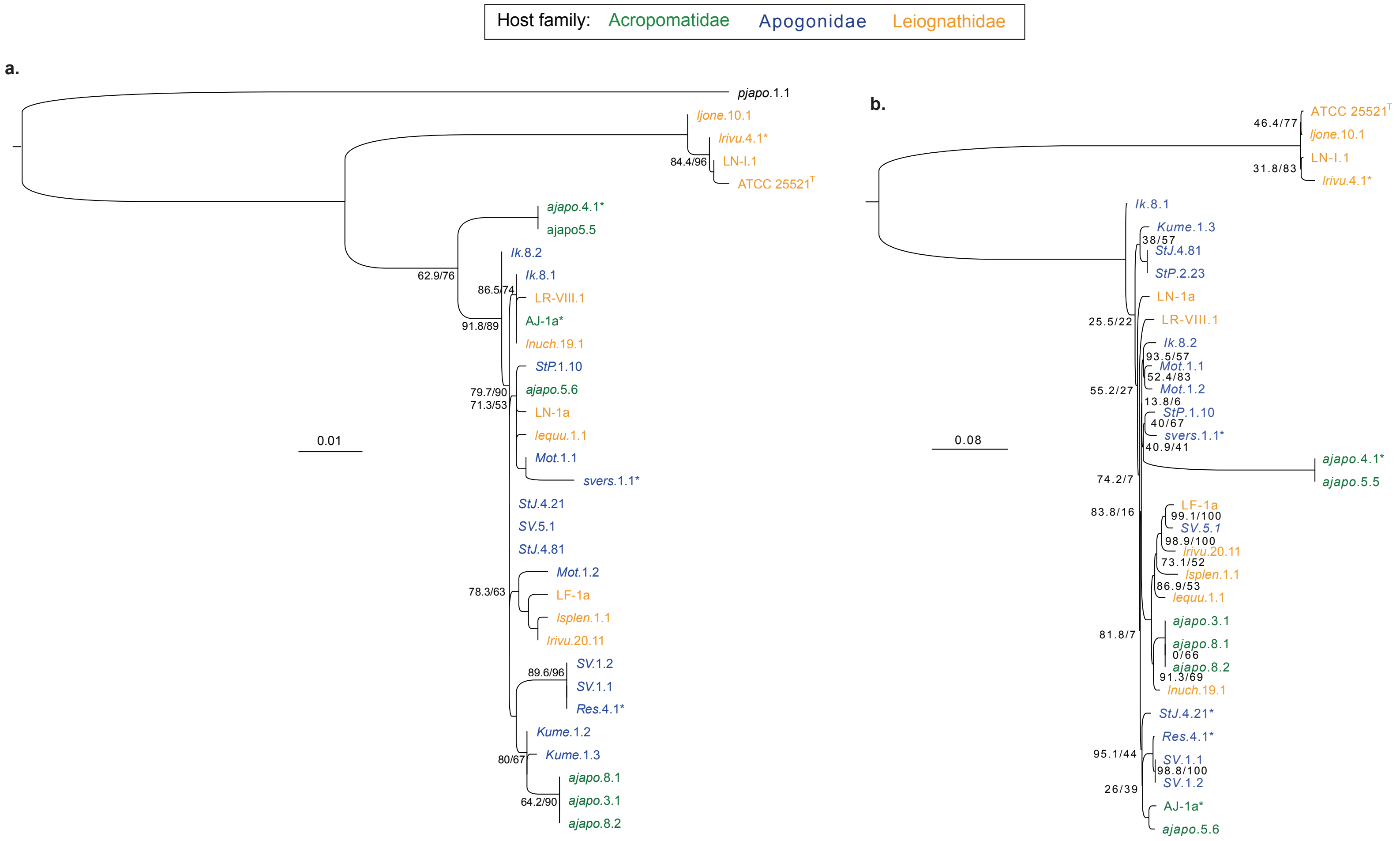

Figure S7. Phylogenetic analysis of *Photobacterium* species isolated from the light organs of various fish hosts based on (a) an alignment of two orthologous genes described in Urbanczyk *et al.* 2013 using the GTR+F+I+G4 model and (b) six genes involved in the type II secretion system (*gspC, gspD, gspJ, gspK, gspL, and gspM*) using the TIM3+F+I+G4 model*.* Midpoint rooted trees are shown for the analysis with 1,000 bootstrap replicates in IQ-TREE. Tip labels are colored according to which family of host fish the strain originated from as indicated in the legend above. Reference strains included in the analyses are indicated by an *. Scale bars show the inferred number of nucleotide substitutions per site. Values listed on branches indicate bootstrap/SH-aLRT support.

Table S1. Summary of draft assemblies before and after scaffolding. Listed are the number of contigs, the total number of bp in the assembly, the largest contig size (bp), the GC content, N50 and L50 values, the number of coding sequences (CDS), the number of rRNAs and tRNAs and the BUSCO scores: complete [single, duplicate], fragmented, and missing.

Strain	Assembly	Ctgs	Total bp	GC%	N50	L50	Ns	CDS	rRNA	tRNA	Complete [S, D]	Fragment	Missing
pjapo.1.1	pre-scaffold	38	4389259	39.36	286117	5	0	5960	25	196	66.1% [64.8%, 1.3%]	12.10%	21.80%
	ragtag scaffold	4	4392659	39.36	3165177	1	77	5932	25	1	66.1% [64.8%, 1.3%]	12.00%	21.90%
	**ragout scaffold**	**4**	**5000498**	**39.36**	**3339696**	**1**	**12224**	**6596**	**25**	**196**	**66.1% [64.8%, 1.3%]**	**12.00%**	**21.90%**
ckamo.1.1	pre-scaffold	27	4913886	38.96	481211	4	0	6183	8	173	74.0% [73.2%, 0.8%]	9.50%	16.50%
	ragtag scaffold	4	4916186	38.96	3232254	1	47	6027	8	173	74.0% [73.2%, 0.8%]	9.50%	16.50%
	**ragout scaffold**	**4**	**5039339**	**38.96**	**3317370**	**1**	**2489**	**6136**	**8**	**173**	**74.0% [73.2%, 0.8%]**	**9.50%**	**16.50%**
ahane.1.5	pre-scaffold	8	5094422	39.02	2927850	1	0	4694	53	207	95.2% [90.5%, 4.7%]	1.60%	3.20%
	ragtag scaffold	5	5094722	39.02	3347557	1	6	4676	53	207	95.2% [90.5%, 4.7%]	1.60%	3.20%
	**ragout scaffold**	**5**	**5100844**	**39.02**	**3353657**	**1**	**126**	**4680**	**53**	**207**	**95.2% [90.5%, 4.7%]**	**1.60%**	**3.20%**
calba.1.1	pre-scaffold	16	5159790	38.96	840622	3	0	4895	46	206	95.0% [94.7%, 0.3%]	1.50%	3.50%
	ragtag scaffold	7	5160690	38.96	3249927	1	17	4880	46	206	95.0% [94.7%, 0.3%]	1.50%	3.50%
	**ragout scaffold**	**7**	**5200973**	**38.96**	**3263220**	**1**	**792**	**4892**	**46**	**206**	**95.0% [94.7%, 0.3%]**	**1.50%**	**3.50%**
Mot.1.1	pre-scaffold	8	4676157	41.25	1938146	2	0	4066	62	208	98.5% [98.1%, 0.4%]	0.20%	1.30%
	**ragtag scaffold**	**2**	**4676757**	**41.25**	**3973505**	**1**	**13**	**4069**	**62**	**208**	**98.5% [98.1%, 0.4%]**	**0.20%**	**1.30%**
	ragout scaffold	7	4676179	41.25	3973427	1	0	4069	62	208	98.5% [98.1%, 0.4%]	0.20%	1.30%
lnuch.19.1	pre-scaffold	8	5229188	41.18	1534541	2	0	4566	57	197	98.8% [98.2%, 0.6%]	0.50%	0.70%
	ragtag scaffold	-	-	-	-	-	-	-	-	-	-	-	-
	**ragout scaffold**	**5**	**5237683**	**41.18**	**3439577**	**1**	**162**	**4618**	**57**	**197**	**98.7% [98.1%, 0.6%]**	**0.50%**	**0.80%**
ajapo.8.2	pre-scaffold	3	4886267	41.15	3261968	1	0	4294	53	194	98.8% [98.3%, 0.5%]	0.30%	0.90%
	ragtag scaffold	3	4886267	41.15	3261968	1	0	4240	53	194	98.8% [98.3%, 0.5%]	0.40%	1.10%
	**ragout scaffold**	**3**	**4886267**	**41.15**	**3261968**	**1**	**0**	**4239**	**53**	**194**	**98.8% [98.3%, 0.5%]**	**0.30%**	**0.90%**
lsplen.1.1	pre-scaffold	14	5249460	41.01	1177766	2	0	4818	37	174	98.3% [97.8%, 0.5%]	0.60%	1.10%
	ragtag scaffold	7	5250160	41.01	3241604	1	13	4756	37	174	98.3% [97.8%, 0.5%]	0.60%	1.60%
	**ragout scaffold**	**7**	**5292468**	**41.01**	**3284001**	**1**	**813**	**4768**	**37**	**174**	**98.3% [97.8%, 0.5%]**	**0.60%**	**1.10%**
Mot.1.2	pre-scaffold	8	4820778	41.2	794793	2	0	4289	62	206	97.3% [97.0%, 0.3%]	1.10%	1.60%
	ragtag scaffold	2	4821378	41.2	3197395	1	12	4317	62	206	97.3% [97.0%, 0.3%]	1.10%	1.60%
	**ragout scaffold**	**2**	**4947312**	**41.2**	**3234211**	**1**	**2558**	**4336**	**62**	**206**	**97.3% [97.0%, 0.3%]**	**1.10%**	**1.60%**

LN-I.1	pre-scaffold	26	5489529	41.53	715204	3	0	4995	62	200	98.0% [97.4%, 0.6%]	0.60%	1.40%
	**ragtag scaffold**	**15**	**5490629**	**41.53**	**3405483**	**1**	**20**	**4994**	**62**	**200**	**98.0% [97.4%, 0.6%]**	**0.60%**	**1.40%**
	ragout scaffold	16	5589437	41.53	3496794	1	1787	5017	62	200	98.0% [97.4%, 0.6%]	0.60%	1.40%
Kume.1.2	pre-scaffold	4	4756280	41.25	3035257	1	0	4208	49	197	98.8% [98.5%, 0.3%]	0.30%	0.90%
	ragtag scaffold	3	4756380	41.25	3186856	1	2	4145	49	197	98.8% [98.5%, 0.3%]	0.60%	1.10%
	**ragout scaffold**	**3**	**4835311**	**41.25**	**3186767**	**1**	**1634**	**4156**	**49**	**197**	**98.8% [98.5%, 0.3%]**	**0.30%**	**0.90%**
SV.1.2	**pre-scaffold**	**3**	**4718342**	**41.17**	**3184478**	**1**	**0**	**4083**	**51**	**200**	**98.5% [98.1%, 0.4%]**	**0.30%**	**1.20%**
	ragtag scaffold	2	4718442	41.17	3184478	1	2	4082	51	200	98.5% [98.1%, 0.4%]	0.30%	1.20%
	ragout scaffold	2	4718353	41.17	3184478	1	0	4083	51	200	98.5% [98.1%, 0.4%]	0.30%	1.20%
Ik.8.1	pre-scaffold	3	4765281	41.15	3231342	1	0	4127	52	202	98.9% [98.6%, 0.3%]	0.20%	0.90%
	ragtag scaffold	3	4765281	41.15	3231342	1	0	4129	47	197	98.9% [98.6%, 0.3%]	0.20%	0.90%
	**ragout scaffold**	**3**	**4765281**	**41.15**	**3231342**	**1**	**0**	**4130**	**47**	**197**	**98.9% [98.6%, 0.3%]**	**0.20%**	**0.90%**
Kume.1.3	pre-scaffold	5	4726851	41.23	2926205	1	0	4177	43	194	98.4% [98.1%, 0.3%]	0.50%	1.10%
	ragtag scaffold	3	4727051	41.23	3172182	1	4	4127	43	194	98.4% [98.1%, 0.3%]	0.50%	1.10%
	**ragout scaffold**	**3**	**4818955**	**41.23**	**3185044**	**1**	**1911**	**4137**	**43**	**194**	**98.4% [98.1%, 0.3%]**	**0.50%**	**1.10%**
StP.2.23	pre-scaffold	38	4202604	41.09	209731	7	0	4219	27	152	77.8% [77.7%, 0.1%]	3.60%	18.60%
	ragtag scaffold	6	4205804	41.09	2658946	1	76	4211	20	144	77.8% [77.7%, 0.1%]	3.60%	18.60%
	**ragout scaffold**	**4**	**4888897**	**41.09**	**3236904**	**1**	**14038**	**4525**	**20**	**144**	**77.8% [77.7%, 0.1%]**	**3.60%**	**18.60%**
StJ.4.21	pre-scaffold	11	4847051	41.23	929383	3	0	4346	63	208	95.8% [95.5%, 0.3%]	1.40%	2.80%
	ragtag scaffold	4	4847751	41.23	3198579	1	14	4351	63	208	95.8% [95.5%, 0.3%]	1.40%	2.80%
	**ragout scaffold**	**4**	**4877430**	**41.23**	**3228436**	**1**	**623**	**4361**	**63**	**208**	**95.8% [95.5%, 0.3%]**	**1.40%**	**2.80%**
SV.1.1	pre-scaffold	8	4687119	41.13	2918729	1	0	4180	38	190	98.1% [97.5%, 0.6%]	0.60%	1.30%
	ragtag scaffold	2	4687719	41.13	3165485	1	13	4116	38	190	98.1% [97.5%, 0.6%]	0.60%	1.30%
	**ragout scaffold**	**2**	**4749844**	**41.13**	**3174417**	**1**	**1321**	**4126**	**38**	**190**	**98.1% [97.5%, 0.6%]**	**0.60%**	**1.30%**
StP.1.10	pre-scaffold	12	4780150	41.15	470369	3	0	4189	47	194	98.8% [98.5%, 0.3%]	0.30%	0.90%
	ragtag scaffold	3	4781050	41.15	3175608	1	19	4194	47	194	98.8% [98.5%, 0.3%]	0.30%	0.90%
	**ragout scaffold**	**4**	**4853990**	**41.15**	**3193844**	**1**	**1521**	**4208**	**47**	**194**	**98.8% [98.5%, 0.3%]**	**0.30%**	**0.90%**
StJ.4.81	pre-scaffold	42	4610923	41.33	215021	6	0	4134	46	190	94.5% [93.3%, 1.2%]	1.60%	3.90%
	ragtag scaffold	3	4614823	41.33	3008273	1	85	4279	46	187	94.6% [93.4%, 1.2%]	1.60%	3.80%
	**ragout scaffold**	**3**	**4967967**	**41.33**	**3223230**	**1**	**7187**	**4378**	**46**	**187**	**94.4% [93.2%, 1.2%]**	**1.60%**	**4.00%**
ajapo.8.1	pre-scaffold	4	4878649	41.12	3245602	1	0	4281	44	190	98.5% [97.9%, 0.6%]	0.70%	0.80%
	ragtag scaffold	4	4878649	41.12	3245602	1	0	4254	44	192	98.5% [97.9%, 0.6%]	0.70%	0.80%

	**ragout scaffold**	**4**	**4878649**	**41.12**	**3245602**	**1**	**0**	**4255**	**44**	**192**	**98.5% [97.9%, 0.6%]**	**0.70%**	**0.80%**
ajapo.5.6	pre-scaffold	2	4729792	41.27	3174233	1	0	4104	62	213	98.7% [98.4%, 0.3%]	0.20%	1.10%
	ragtag scaffold	2	4729792	41.27	3174233	1	0	4052	62	213	98.7% [98.4%, 0.3%]	0.20%	1.10%
	**ragout scaffold**	**2**	**4729792**	**41.27**	**3174233**	**1**	**0**	**4052**	**62**	**213**	**98.7% [98.4%, 0.3%]**	**0.20%**	**1.10%**
LR-VIII.1	pre-scaffold	105	4467508	41.28	64254	20	0	4593	32	147	76.6% [76.3%, 0.3%]	4.40%	19.00%
	ragtag scaffold	-	-	-	-	-	-	-	-	-	-	-	-
	**ragout scaffold**	**21**	**5791416**	**41.28**	**3699906**	**1**	**22860**	**5602**	**32**	**147**	**76.6% [76.3%, 0.3%]**	**4.20%**	**19.20%**
SV.5.1	pre-scaffold	43	4516983	41.27	190888	8	0	4123	35	193	94.6% [94.3%, 0.3%]	1.20%	4.20%
	ragtag scaffold	2	4521083	41.27	3089627	1	91	4122	35	193	94.7% [94.4%, 0.3%]	1.20%	4.10%
	**ragout scaffold**	**3**	**5111110**	**41.27**	**3324477**	**1**	**11624**	**4280**	**35**	**193**	**94.6% [94.3%, 0.3%]**	**1.20%**	**4.20%**
ljone.10.1	pre-scaffold	9	5276659	41.34	3074291	1	0	4551	62	207	98.7% [98.3%, 0.4%]	0.10%	1.20%
	ragtag scaffold	-	-	-	-	-	-	-	-	-	-	-	-
	**ragout scaffold**	**8**	**5276714**	**41.34**	**3313411**	**1**	**1**	**4535**	**62**	**207**	**98.7% [98.3%, 0.4%]**	**0.10%**	**1.20%**
LN-1a	pre-scaffold	38	5171490	41.21	359929	5	0	4666	44	197	96.1% [95.8%, 0.3%]	1.00%	2.90%
	**ragtag scaffold**	**10**	**5174290**	**41.21**	**3359026**	**1**	**54**	**4760**	**44**	**195**	**96.0% [95.7%, 0.3%]**	**1.10%**	**2.90%**
	ragout scaffold	12	5578355	41.21	3411149	1	7294	4939	44	195	96.1% [95.8%, 0.3%]	1.00%	2.90%
ATCC 25521^{T}	**pre-scaffold**	**3**	**4750881**	**41.02**	**3269131**	**1**	**0**	**4257**	**47**	**199**	**96.3% [95.9%, 0.4%]**	**1.60%**	**2.10%**
	ragtag scaffold	3	4750881	41.02	3269131	1	0	4254	41	194	96.3% [95.9%, 0.4%]	1.70%	2.10%
	ragout scaffold	3	4750892	41.02	3269131	1	0	4253	41	194	96.3% [95.9%, 0.4%]	1.60%	2.10%
lequu.1.1	**pre-scaffold**	**4**	**4825624**	**41.19**	**3204041**	**1**	**0**	**4206**	**62**	**209**	**98.7% [98.3%, 0.4%]**	**0.30%**	**1.00%**
	ragtag scaffold	-	-	-	-	-	-	-	-	-	-	-	-
	ragout scaffold	4	4825624	41.19	3204041	1	0	4192	62	209	98.7% [98.3%, 0.4%]	0.30%	1.00%
LF-1a	pre-scaffold	13	5042264	40.96	915751	2	0	4575	36	189	98.5% [97.9%, 0.6%]	0.50%	1.00%
	ragtag scaffold	6	5042964	40.96	3186469	1	14	4593	39	192	98.5% [97.9%, 0.6%]	1.80%	2.70%
	**ragout scaffold**	**5**	**5104659**	**40.96**	**3267821**	**1**	**1222**	**4607**	**39**	**192**	**98.5% [97.9%, 0.6%]**	**0.50%**	**1.00%**

Table S2. Pairwise ANI values between all *Photobacterium* genomes assembled in this study. Additional reference strains included for comparison are indicated by an *.

	**_pjapo_.1.1**^{T}	**_ckamo_.1.1**	**_ahane_.1.5**	**_calba_.1.1**	**_pjapo_.1.1**^{T}*****	**_ajapo.5.5_**	**_ajapo.4.1*_**	**LR-VIII.1**	**_ajapo.3.1*_**	**_ajapo.8.2_**
**_pjapo_.1.1**^{T}	100	97.32	97.54	97.36	97.64	80.70	80.25	80.79	80.37	80.83
**_ckamo_.1.1**	97.32	100	98.68	98.55	99.47	80.69	80.43	80.87	80.40	80.71
**_ahane_.1.5**	97.54	98.68	100	98.70	99.04	80.74	80.23	80.77	80.26	80.67
**_calba_.1.1**	97.36	98.55	98.70	100	98.91	80.51	80.34	80.43	80.33	80.45
**_pjapo_.1.1**^{T}*****	97.64	99.47	99.04	98.91	100	80.20	80.19	80.36	80.32	80.30
**_ajapo_.5.5**	80.70	80.69	80.74	80.51	80.20	100	99.98	92.85	92.89	92.96
**_ajapo_.4.1***	80.25	80.43	80.23	80.34	80.19	99.98	100	92.76	92.86	92.87
**LR-VIII.1**	80.79	80.87	80.77	80.43	80.36	92.85	92.76	100	96.24	96.29
**_ajapo_.3.1***	80.37	80.40	80.26	80.33	80.32	92.89	92.86	96.24	100	99.96
**_ajapo_.8.2**	80.83	80.71	80.67	80.45	80.30	92.96	92.87	96.29	99.96	100
**_ajapo_.8.1**	80.83	80.76	80.69	80.51	80.32	92.98	92.87	96.31	99.96	99.96
**LN-I.1**	80.74	80.72	80.69	80.39	80.15	93.15	93.07	95.85	96.45	96.45
**_lrivu_.4.1***	80.30	80.42	80.17	80.28	80.27	93.08	93.02	95.65	96.46	96.39
**_ljone_.10.1**	80.69	80.66	80.70	80.50	80.19	93.01	92.95	95.95	96.60	96.64
**ATCC 25521**^{T}	80.79	80.89	80.75	80.57	80.33	93.02	92.92	95.96	96.51	96.54
**LN-1a**	80.84	80.71	80.68	80.45	80.27	92.97	92.85	96.73	96.70	96.77
**_StP_.2.23**	80.31	80.28	80.17	80.12	79.93	92.53	92.56	96.08	96.75	96.73
**_Kume_.1.2**	80.59	80.72	80.55	80.31	80.27	92.95	92.82	96.51	97.12	97.08
**_Kume_.1.3**	80.62	80.59	80.66	80.34	80.22	92.92	92.88	96.46	97.09	97.10
**_Res_.4.1**	80.28	80.38	80.21	80.20	80.13	92.82	92.83	96.45	97.09	97.07
**_SV_.1.1**	80.70	80.71	80.61	80.36	80.21	92.86	92.78	96.48	97.11	97.12
**_SV_.1.2**	80.87	80.77	80.74	80.47	80.25	92.90	92.80	96.49	97.11	97.16
**AJ-1**	80.17	80.39	80.22	80.30	80.16	92.96	92.96	96.56	97.03	97.04
**_lnuch_.19.1**	80.74	80.78	80.63	80.43	80.23	92.89	92.85	96.50	97.07	97.10

**_lrivu_.20.11**	80.85	80.79	80.67	80.42	80.32	92.95	92.84	96.56	97.12	97.15
**_ajapo_.5.6**	80.85	80.78	80.82	80.49	80.36	93.09	92.96	96.66	97.11	97.15
**_SV_.5.1**	80.62	80.78	80.63	80.42	80.27	92.91	92.87	96.40	96.98	96.98
**_StJ_.4.81**	80.73	80.60	80.55	80.32	80.24	92.84	92.78	96.36	96.98	97.02
**_svers_.1.1***	80.30	80.34	80.15	80.25	80.17	92.83	92.83	96.48	97.12	97.09
**_StJ_.4.21**	80.82	80.75	80.70	80.47	80.25	92.81	92.79	96.34	96.95	96.98
**_Mot_.1.1**	80.91	80.81	80.77	80.42	80.30	92.96	92.86	96.42	97.02	97.07
**_Mot_.1.2**	80.83	80.64	80.73	80.35	80.20	92.92	92.82	96.43	97.00	97.05
**_StP_.1.10**	80.65	80.65	80.69	80.42	80.14	92.87	92.85	96.47	97.05	97.08
**_lk_.8.2**	80.85	80.75	80.78	80.43	80.31	92.93	92.83	96.60	97.13	97.17
**_lk_.8.1**	80.63	80.63	80.59	80.34	80.18	92.82	92.73	96.48	97.07	97.09
**_lsplen_.1.1**	80.61	80.75	80.56	80.29	80.22	92.89	92.81	96.29	96.90	96.95
**_lequu_.1.1**	80.71	80.68	80.58	80.34	80.14	92.97	92.80	96.34	96.98	97.04
**LF-1a**	80.71	80.72	80.70	80.48	80.32	92.97	92.86	96.31	97.11	97.18

	**_ajapo_.8.1**	**LN-I.1**	**_lrivu_.4.1***	**_ljone_.10.1**	**ATCC 25521**	**LN-1a**	**_StP_.2.23**	**_Kume_.1.2**	**_Kume_.1.3**	**_Res_.4.1**
**_pjapo_.1.1^{T}**	80.83	80.74	80.30	80.69	80.79	80.84	80.31	80.59	80.62	80.28
**_ckamo_.1.1**	80.76	80.72	80.42	80.66	80.89	80.71	80.28	80.72	80.59	80.38
**_ahane_.1.5**	80.69	80.69	80.17	80.70	80.75	80.68	80.17	80.55	80.66	80.21
**_calba_.1.1**	80.51	80.39	80.28	80.50	80.57	80.45	80.12	80.31	80.34	80.20
**_pjapo_.1.1^{T}***	80.32	80.15	80.27	80.19	80.33	80.27	79.93	80.27	80.22	80.13
**_ajapo_.5.5**	92.98	93.15	93.08	93.01	93.02	92.97	92.53	92.95	92.92	92.82
**_ajapo_.4.1***	92.87	93.07	93.02	92.95	92.92	92.85	92.56	92.82	92.88	92.83
**LR-VIII.1**	96.31	95.85	95.65	95.95	95.96	96.73	96.08	96.51	96.46	96.45
**_ajapo_.3.1***	99.96	96.45	96.46	96.60	96.51	96.70	96.75	97.12	97.09	97.09
**_ajapo_.8.2**	99.96	96.45	96.39	96.64	96.54	96.77	96.73	97.08	97.10	97.07
**_ajapo_.8.1**	100	96.48	96.41	96.65	96.56	96.74	96.74	97.08	97.09	97.08
**LN-I.1**	96.48	100	97.85	97.10	97.40	96.22	96.26	96.59	96.61	96.56

Irivu.4.1*	96.41	97.85	100	96.98	97.26	96.11	96.21	96.54	96.56	96.53
Ijone.10.1	96.65	97.10	96.98	100	97.39	96.33	96.46	96.78	96.80	96.78
ATCC 25521^{T}	96.56	97.40	97.26	97.39	100	96.33	96.33	96.66	96.67	96.61
LN-1a	96.74	96.22	96.11	96.33	96.33	100	96.52	96.83	96.87	96.88
StP.2.23	96.74	96.26	96.21	96.46	96.33	96.52	100	97.01	97.01	97.01
Kume.1.2	97.08	96.59	96.54	96.78	96.66	96.83	97.01	100	99.96	97.33
Kume.1.3	97.09	96.61	96.56	96.80	96.67	96.87	97.01	99.96	100	97.35
Res.4.1	97.08	96.56	96.53	96.78	96.61	96.88	97.01	97.33	97.35	100
SV.1.1	97.09	96.57	96.55	96.76	96.68	96.91	96.97	97.37	97.34	98.17
SV.1.2	97.11	96.60	96.56	96.78	96.68	96.99	97.01	97.41	97.40	98.22
AJ-1	97.03	96.48	96.44	96.60	96.50	97.14	96.73	97.07	97.06	97.06
Inuch.19.1	97.07	96.41	96.27	96.63	96.54	96.89	96.83	97.17	97.17	97.18
Irivu.20.11	97.14	96.67	96.59	96.79	96.75	97.06	96.81	97.19	97.22	97.22
ajapo.5.6	97.11	96.61	96.57	96.75	96.61	97.18	96.84	97.19	97.19	97.18
SV.5.1	97.00	96.53	96.45	96.68	96.62	96.82	96.82	97.21	97.21	97.15
StJ.4.81	97.00	96.54	96.42	96.71	96.54	96.77	96.91	97.27	97.27	97.22
svers.1.1*	97.10	96.64	96.64	96.81	96.70	96.89	97.01	97.36	97.35	97.33
StJ.4.21	96.98	96.52	96.43	96.66	96.64	96.80	96.96	97.37	97.34	97.36
Mot.1.1	97.08	96.59	96.55	96.66	96.63	96.87	96.98	97.34	97.30	97.33
Mot.1.2	97.05	96.55	96.57	96.81	96.60	96.86	97.01	97.34	97.36	97.32
StP.1.10	97.08	96.64	96.52	96.78	96.75	96.84	97.05	97.39	97.38	97.34
Ik.8.2	97.14	96.64	96.59	96.84	96.65	96.89	96.99	97.44	97.44	97.39
Ik.8.1	97.07	96.61	96.56	96.79	96.62	96.87	96.97	97.39	97.39	97.38
Isplen.1.1	96.94	96.40	96.19	96.55	96.53	96.71	96.77	97.14	97.14	97.12
Iequu.1.1	97.02	96.60	96.61	96.74	96.66	96.83	96.81	97.14	97.13	97.11
LF-1a	97.15	96.55	96.53	96.62	96.55	96.72	96.69	97.04	97.04	97.02
	SV.1.1	*SV*.1.2	AJ-1	*Inuch*.19.1	*Irivu*.20.11	*ajapo*.5.6	*SV*.5.1	*StJ*.4.81	*svers*.1.1*	*StJ*.4.21

***pjapo*.1.1^{T}**	80.70	80.87	80.17	80.74	80.85	80.85	80.62	80.73	80.30	80.82
***ckamo*.1.1**	80.71	80.77	80.39	80.78	80.79	80.78	80.78	80.60	80.34	80.75
***ahane*.1.5**	80.61	80.74	80.22	80.63	80.67	80.82	80.63	80.55	80.15	80.70
***calba*.1.1**	80.36	80.47	80.30	80.43	80.42	80.49	80.42	80.32	80.25	80.47
pjapo*.1.1^{T}	80.21	80.25	80.16	80.23	80.32	80.36	80.27	80.24	80.17	80.25
***ajapo*.5.5**	92.86	92.90	92.96	92.89	92.95	93.09	92.91	92.84	92.83	92.81
ajapo*.4.1	92.78	92.80	92.96	92.85	92.84	92.96	92.87	92.78	92.83	92.79
**LR-VIII.1**	96.48	96.49	96.56	96.50	96.56	96.66	96.40	96.36	96.48	96.34
ajapo*.3.1	97.11	97.11	97.03	97.07	97.12	97.11	96.98	96.98	97.12	96.95
***ajapo*.8.2**	97.12	97.16	97.04	97.10	97.15	97.15	96.98	97.02	97.09	96.98
***ajapo*.8.1**	97.09	97.11	97.03	97.07	97.14	97.11	97.00	97.00	97.10	96.98
**LN-I.1**	96.57	96.60	96.48	96.41	96.67	96.61	96.53	96.54	96.64	96.52
lrivu*.4.1	96.55	96.56	96.44	96.27	96.59	96.57	96.45	96.42	96.64	96.43
***ljone*.10.1**	96.76	96.78	96.60	96.63	96.79	96.75	96.68	96.71	96.81	96.66
**ATCC 25521^{T}**	96.68	96.68	96.50	96.54	96.75	96.61	96.62	96.54	96.70	96.64
**LN-1a**	96.91	96.99	97.14	96.89	97.06	97.18	96.82	96.77	96.89	96.80
***StP*.2.23**	96.97	97.01	96.73	96.83	96.81	96.84	96.82	96.91	97.01	96.96
***Kume*.1.2**	97.37	97.41	97.07	97.17	97.19	97.19	97.21	97.27	97.36	97.37
***Kume*.1.3**	97.34	97.40	97.06	97.17	97.22	97.19	97.21	97.27	97.35	97.34
***Res*.4.1**	98.17	98.22	97.06	97.18	97.22	97.18	97.15	97.22	97.33	97.36
***SV*.1.1**	100	99.92	97.06	97.16	97.21	97.20	97.11	97.19	97.30	97.62
***SV*.1.2**	99.92	100	97.11	97.21	97.23	97.20	97.16	97.24	97.32	97.67
**AJ-1**	97.06	97.11	100	97.10	97.32	97.29	96.90	96.93	97.09	96.99
***lnuch*.19.1**	97.16	97.21	97.10	100	97.27	97.20	97.10	97.09	97.22	97.15
***lrivu*.20.11**	97.21	97.23	97.32	97.27	100	97.32	97.05	97.07	97.15	97.16
***ajapo*.5.6**	97.20	97.20	97.29	97.20	97.32	100	97.05	97.08	97.15	97.14
***SV*.5.1**	97.11	97.16	96.90	97.10	97.05	97.05	100	97.05	97.16	97.14
***StJ*.4.81**	97.19	97.24	96.93	97.09	97.07	97.08	97.05	100	97.23	97.20

*svers.*1.1*	97.30	97.32	97.09	97.22	97.15	97.15	97.16	97.23	100	97.28
*StJ.*4.21	97.62	97.67	96.99	97.15	97.16	97.14	97.14	97.20	97.28	100
*Mot.*1.1	97.38	97.36	97.06	97.19	97.16	97.18	97.11	97.21	97.25	97.30
*Mot.*1.2	97.30	97.32	97.03	97.21	97.18	97.15	97.13	97.21	97.29	97.28
*StP.*1.10	97.33	97.40	97.07	97.19	97.20	97.20	97.17	97.26	97.34	97.31
*Ik.*8.2	97.44	97.44	97.09	97.23	97.23	97.15	97.20	97.30	97.34	97.36
*Ik.*8.1	97.28	97.35	97.12	97.17	97.19	97.16	97.19	97.23	97.30	97.29
*Isplen.*1.1	97.13	97.16	96.97	97.02	97.12	97.09	97.00	96.98	97.13	97.03
*Iequu.*1.1	97.10	97.14	97.05	97.18	97.18	97.16	97.07	97.03	97.09	97.12
LF-1a	97.03	97.10	96.96	97.03	97.10	97.04	97.00	96.91	97.02	96.99

	*Mot.*1.1	*Mot.*1.2	*StP.*1.10	*Ik.*8.2	*Ik.*8.1	*Isplen.*1.1	*Iequu.*1.1	LF-1a
*pjapo.*1.1[T]	80.91	80.83	80.65	80.85	80.63	80.61	80.71	80.71
*ckamo.*1.1	80.81	80.64	80.65	80.75	80.63	80.75	80.68	80.72
*ahane.*1.5	80.77	80.73	80.69	80.78	80.59	80.56	80.58	80.70
*calba.*1.1	80.42	80.35	80.42	80.43	80.34	80.29	80.34	80.48
*pjapo.*1.1[T]*	80.30	80.20	80.14	80.31	80.18	80.22	80.14	80.32
*ajapo.*5.5	92.96	92.92	92.87	92.93	92.82	92.89	92.97	92.97
*ajapo.*4.1*	92.86	92.82	92.85	92.83	92.73	92.81	92.80	92.86
LR-VIII.1	96.42	96.43	96.47	96.60	96.48	96.29	96.34	96.31
*ajapo.*3.1*	97.02	97.00	97.05	97.13	97.07	96.90	96.98	97.11
*ajapo.*8.2	97.07	97.05	97.08	97.17	97.09	96.95	97.04	97.18
*ajapo.*8.1	97.08	97.05	97.08	97.14	97.07	96.94	97.02	97.15
LN-I.1	96.59	96.55	96.64	96.64	96.61	96.40	96.60	96.55
*lrivu.*4.1*	96.55	96.57	96.52	96.59	96.56	96.19	96.61	96.53
*ljone.*10.1	96.66	96.81	96.78	96.84	96.79	96.55	96.74	96.62
ATCC 25521[T]	96.63	96.60	96.75	96.65	96.62	96.53	96.66	96.55
LN-1a	96.87	96.86	96.84	96.89	96.87	96.71	96.83	96.72

***StP*.2.23**	96.98	97.01	97.05	96.99	96.97	96.77	96.81	96.69
***Kume*.1.2**	97.34	97.34	97.39	97.44	97.39	97.14	97.14	97.04
***Kume*.1.3**	97.30	97.36	97.38	97.44	97.39	97.14	97.13	97.04
***Res*.4.1**	97.33	97.32	97.34	97.39	97.38	97.12	97.11	97.02
***SV*.1.1**	97.38	97.30	97.33	97.44	97.28	97.13	97.10	97.03
***SV*.1.2**	97.36	97.32	97.40	97.44	97.35	97.16	97.14	97.10
**AJ-1**	97.06	97.03	97.07	97.09	97.12	96.97	97.05	96.96
***Inuch*.19.1**	97.19	97.21	97.19	97.23	97.17	97.02	97.18	97.03
***Irivu*.20.11**	97.16	97.18	97.20	97.23	97.19	97.12	97.18	97.10
***ajapo*.5.6**	97.18	97.15	97.20	97.15	97.16	97.09	97.16	97.04
***SV*.5.1**	97.11	97.13	97.17	97.20	97.19	97.00	97.07	97.00
***StJ*.4.81**	97.21	97.21	97.26	97.30	97.23	96.98	97.03	96.91
svers*.1.1	97.25	97.29	97.34	97.34	97.30	97.13	97.09	97.02
***StJ*.4.21**	97.30	97.28	97.31	97.36	97.29	97.03	97.12	96.99
***Mot*.1.1**	100	97.27	97.33	97.33	97.38	97.12	97.13	97.00
***Mot*.1.2**	97.27	100	97.36	97.38	97.33	97.16	97.10	97.01
***StP*.1.10**	97.33	97.36	100	97.39	97.37	97.17	97.21	97.04
***Ik*.8.2**	97.33	97.38	97.39	100	97.42	97.17	97.20	97.04
***Ik*.8.1**	97.38	97.33	97.37	97.42	100	97.16	97.11	97.02
***Isplen*.1.1**	97.12	97.16	97.17	97.17	97.16	100	97.14	96.98
***Iequu*.1.1**	97.13	97.10	97.21	97.20	97.11	97.14	100	97.20
**LF-1a**	97.00	97.01	97.04	97.04	97.02	96.98	97.20	100

Table S3. Comparison of the long read-only and hybrid draft assemblies for the two *P. 'mandapamensis'* strains for which short reads were available. Listed are the number of contigs (Ctgs), the total number of bp in the assembly, the largest contig size (bp), the GC content, N50 and L50 values, the average number of Ns/100 kbp, the number of coding sequences (CDS), the number of rRNAs and tRNAs, the number of repeat regions, and the BUSCO scores shown as percentages: complete [single, duplicate], fragmented, and missing. The final assembly selected for analysis is indicated in bold.

Strain	Assembly	Ctgs	Total bp	GC%	N50	L50	Ns	CDS	rRNA	tRNA	Repeat	Complete [S, D]	Fragment	Missing
StP.2.23	flye+circ+polish	38	4228014	41.09	209731	7	0	4211	20	144	0	77.8 [77.7, 0.1]	3.60	18.60
	flye+circ+polish+scaffold	4	4888897	41.09	3236904	1	14038	4525	20	144	0	77.8 [77.7, 0.1]	3.60	18.60
	unicycler	30	4686987	41.18	2741469	1	0	4044	33	188	2	99.1 [98.8, 0.3]	0.10	0.80
	unicycler+circ	4	4688278	41.18	3000960	1	0	4112	35	182	2	96.0 [95.7, 0.3]	1.20	2.80
	unicycler+circ+scaffold	2	4688478	41.18	3134673	1	4.27	4112	35	182	2	96.0 [95.7, 0.3]	1.20	2.80
	unicycler+scaffold	25	4691567	41.18	3130376	1	10.67	4044	33	188	2	98.5 [98.0, 0.5]	0.20	1.30
	**unicycler+scaffold+circ**	**2**	**4689350**	**41.18**	**3135545**	**1**	**4.26**	**4095**	**35**	**181**	**2**	**96.3 [96.0, 0.3]**	**1.00**	**2.70**
StJ.4.81	flye+circ+polish	42	4610923	41.33	215021	6	0	4280	46	187	0	94.5 [93.3, 1.2]	1.60	3.90
	flye+circ+polish+scaffold	3	4614823	41.33	3008273	1	84.51	4279	46	187	0	94.6 [93.4, 1.2]	1.60	3.80
	unicycler	15	4716676	41.23	1555055	2	0	4047	45	196	2	99.1 [98.8, 0.3]	0.10	0.80
	unicycler+circ	2	4716071	41.23	3161731	1	0	4160	47	198	2	95.9 [95.6, 0.3]	1.00	3.10
	unicycler+circ+scaffold	2	4716071	41.23	3161731	1	0	4161	47	198	2	95.9 [95.6, 0.3]	1.00	3.10
	unicycler+scaffold	12	4716976	41.23	3157280	1	6.36	4047	45	196	2	99.1 [98.8, 0.3]	0.10	0.80
	**unicycler+scaffold+circ**	**2**	**4711728**	41.22	3157280	1	6.37	**4101**	**44**	**195**	**2**	**98.6 [98.3, 0.3]**	**0.10**	**1.30**

Table S4. Genes present in plasmid sequences of the *Photobacterium* strains indicated and their copy number detected. Reference sequences from NCBI included in the analysis are indicated with a *.

gene	ajapo4.1*	ajapo5.5	ajapo8.2	ATCC25521	Ik8.1	Ik8.2	Kume1.2	Kume1.3	JS01*	Inuch19.1	Isplen1.1
accD	0	0	0	0	0	0	0	0	0	0	0
Acetyltransferase	0	0	0	0	0	0	0	0	0	0	1
adhB	0	0	0	0	1	1	0	0	1	2	1
aguA	0	0	0	0	0	0	0	0	0	0	0
argO	0	0	0	0	0	0	0	0	1	0	1
bin3	1	1	1	0	0	1	0	0	1	1	1
bioF	0	0	0	0	0	0	0	0	0	0	0
btr	0	0	0	0	0	0	0	0	0	0	0
btuD	0	0	0	0	0	0	0	0	0	0	0
cas1	0	0	0	0	0	0	0	0	0	0	0
cas2	0	0	0	0	0	0	0	0	0	0	0
cobS	0	0	0	0	0	0	0	0	0	0	0
copR	0	0	0	0	0	0	0	0	0	0	0
cpdA	0	0	0	0	0	0	0	0	0	0	0
cspG	0	0	0	0	0	0	0	0	0	1	0
cyaB	0	0	0	0	0	0	0	0	0	0	0
cyt1Aa	0	0	0	0	0	0	0	0	1	0	0
dapH	0	0	0	0	0	0	0	0	0	0	1
ddl	0	0	0	0	0	0	0	0	0	0	1
degP	0	0	0	0	0	0	0	0	0	0	0
degQ	0	0	0	0	0	0	0	0	0	0	0
dinB	0	0	0	2	0	0	0	0	0	0	0
dnaG	0	0	0	0	0	0	0	0	0	0	1
dns	1	1	0	0	0	0	2	0	0	1	2
dosP	0	0	0	0	0	0	0	0	0	0	0
echA8	0	0	0	0	0	0	0	0	0	0	0
elaA	0	0	0	0	0	0	0	0	0	0	1
elmMIII	0	0	0	0	0	0	0	0	0	0	1
emrE	0	0	0	0	0	0	0	0	0	1	1
epsE	0	0	0	0	0	0	0	0	0	0	1
exsA	0	0	0	0	0	0	0	0	0	0	0
Extracellular serine proteinase	0	0	0	0	0	0	0	0	0	0	0
Ferredoxin--NADP reductase	0	0	0	0	0	0	0	0	0	0	0
ghrA	0	0	0	0	0	0	0	0	0	0	1
gmk	0	0	0	0	0	0	0	0	0	0	0
groL	0	0	0	0	0	0	0	0	0	0	1
groS	0	0	0	0	0	0	0	0	0	0	1

gst	0	0	0	0	0	0	0	0	0	0	1
hapE	0	0	0	0	0	0	0	0	0	0	0
higA1	0	0	0	0	0	0	0	0	0	0	0
hipA	0	0	0	0	0	1	0	0	0	0	0
hisC2	0	0	0	0	0	0	0	0	0	0	1
hns	0	0	0	0	0	0	0	0	0	1	1
hsdR	0	0	0	0	0	0	0	0	0	0	0
htrC	0	0	0	0	0	0	0	0	0	0	0
hup	0	0	0	0	0	0	0	0	0	0	1
hxpA	0	0	0	0	0	0	0	0	0	0	0
ilvE	0	0	0	0	0	0	0	0	0	0	1
imm	0	0	0	0	0	0	0	0	0	0	0
invA	0	0	0	0	0	0	0	0	0	0	0
IS1 family transposase ISPda1	0	0	0	0	0	0	0	0	0	0	0
IS110 family transposase ISCps1	0	0	0	0	0	0	0	0	0	0	1
IS110 family transposase ISSde4	1	0	1	0	0	0	0	0	0	0	0
IS110 family transposase ISSpi6	0	0	0	0	0	0	0	0	0	0	0
IS200/IS605 family transposase ISEc42	0	0	0	0	0	0	0	0	0	0	0
IS200/IS605 family transposase ISEc46	0	0	0	0	0	0	0	0	0	0	0
IS21 family transposase ISVch3	0	0	0	0	0	0	0	0	0	0	0
IS3 family transposase ISBps2	0	0	0	0	0	0	0	0	0	0	0
IS3 family transposase ISThsp4	0	0	0	0	0	0	0	0	0	0	0
IS3 family transposase ISVch4	0	0	0	0	0	0	0	0	0	0	0
IS30 family transposase ISSde3	0	0	0	0	0	0	0	0	0	0	0
IS4 family transposase ISAzvi5	0	0	0	0	0	0	0	0	0	0	0
IS4 family transposase ISCro6	0	0	0	0	0	0	0	0	0	0	0
IS4 family transposase ISPcc6	0	0	0	0	0	0	0	0	0	0	0
IS4 family transposase ISVa18	0	0	0	0	0	0	0	0	0	0	0
IS4 family transposase ISVsa5	0	0	0	0	0	0	0	0	0	0	0
IS5 family transposase ISVha3	0	0	0	0	0	0	0	0	0	0	0
IS6 family transposase ISPpr9	0	0	1	0	0	0	0	0	2	6	11
IS630 family transposase ISVa15	0	0	1	0	0	0	0	0	0	0	1
IS66 family transposase ISVa11	0	0	0	0	0	0	0	0	0	1	0
IS66 family transposase ISVa5	0	0	0	0	0	0	0	0	0	0	0
IS66 family transposase ISVa9	0	0	0	0	0	0	0	0	0	0	0
IS91 family transposase ISShvi3	0	0	0	0	0	0	0	0	0	0	1
IS91 family transposase ISVsa9	0	0	0	0	0	0	0	0	0	0	1
ISAs1 family transposase ISVha1	0	0	0	0	0	0	0	0	0	0	0
Isatin hydrolase	0	0	0	0	0	0	0	0	0	0	1
ISNCY family transposase ISLad2	0	0	0	0	0	0	0	0	0	0	0
ISNCY family transposase ISLad2	0	0	0	0	0	0	0	0	0	0	0
ISNCY family transposase ISVisp7	0	0	0	0	0	0	0	0	0	0	0

kynA	0	0	0	0	0	0	0	0	0	0	1
kynu	0	0	0	0	0	0	0	0	0	0	1
lagD	0	0	0	0	1	1	0	0	0	1	0
lcdH	0	0	0	0	0	0	0	0	0	0	2
lgrD	0	0	0	0	0	0	0	0	0	0	0
lysO	0	0	0	0	0	0	0	0	0	0	0
mhpA	0	0	0	0	0	0	0	0	0	0	1
moaA	0	0	0	0	0	0	0	0	0	0	0
mtcA2	0	0	0	0	0	0	0	0	0	0	0
N-carbamoyl-D-amino acid hydrolase	0	0	0	0	0	0	0	0	0	0	0
nimR	2	0	2	0	0	0	0	0	0	0	0
nrgA	0	0	0	0	0	0	0	0	0	0	0
ntdA	0	0	0	0	0	0	0	0	0	0	0
ntdB	0	0	0	0	0	0	0	0	0	0	0
ntdC	0	0	0	0	0	0	0	0	0	0	0
nucM	0	0	0	0	0	0	0	0	0	0	0
ompA	0	0	0	0	0	0	0	0	0	0	1
parA	2	0	0	0	0	0	0	0	0	0	0
pdeG	0	0	0	0	0	0	0	0	0	0	0
pgrR	0	0	0	0	0	0	0	0	0	0	1
pinR	0	0	0	0	1	0	2	1	0	0	3
pndA	0	0	0	0	0	0	0	0	0	0	1
proQ	0	0	0	0	0	0	0	0	0	0	0
prtR	0	0	0	0	0	0	0	0	0	0	1
pscF	0	0	0	0	0	0	0	0	0	0	0
ptlH	0	0	0	0	0	0	0	0	0	0	1
Putative nickel-responsive regulator	0	0	0	0	0	0	0	0	0	0	0
putative oxidoreductase	0	0	0	0	0	0	0	0	0	0	1
queuosine precursor transporter	0	0	0	0	0	0	0	0	0	1	0
ramA	0	0	0	0	0	0	0	0	1	0	0
ramC	0	0	0	0	0	0	0	0	0	1	0
rclC	0	0	0	0	0	0	0	0	0	0	1
recX	0	0	0	0	0	0	0	0	0	0	0
rep	0	0	0	0	0	0	0	0	0	0	0
repA	1	1	1	0	0	0	2	1	0	1	1
rhaR	0	0	0	0	0	0	0	0	0	1	0
rhaS	0	0	0	0	0	0	0	0	0	0	1
rhsC	0	0	0	0	0	0	0	0	0	1	0
ribD	0	0	0	0	0	0	0	0	0	0	0
ribF	0	0	0	0	0	0	0	0	0	1	0
rizA	0	0	0	0	0	0	0	0	0	0	1
rutB	0	0	0	0	0	0	0	0	1	0	1

sasA	0	0	0	0	0	0	0	0	0	0	0
sctC	0	0	0	0	0	0	0	0	0	0	0
slyA	0	0	0	0	1	1	0	0	0	0	0
smc	0	0	0	0	0	1	0	0	0	0	0
sodC	0	0	0	0	0	0	0	0	0	0	1
soj	0	0	0	0	0	0	0	0	0	0	0
soxR	0	0	0	0	0	0	0	0	0	0	0
spaR	0	0	0	0	0	0	0	0	0	0	0
tcpE	0	0	0	0	0	0	0	0	0	0	0
TDO2	0	0	0	0	0	0	0	0	0	0	1
tetA	0	0	0	0	0	0	0	0	0	0	1
tfdR	0	0	0	0	0	0	0	0	1	0	0
tmcA	2	0	0	0	0	0	0	0	0	0	0
Tn3 family transposase ISSod9	0	0	0	0	0	0	0	0	0	0	0
Tn3 family transposase ISXc4	0	0	0	0	0	0	0	0	0	0	0
tnpR	1	1	1	1	1	0	2	1	1	1	2
topA	0	0	0	0	0	0	0	0	0	0	0
topB	0	0	0	0	0	0	0	0	0	0	1
traC	0	0	0	0	0	0	0	0	0	1	2
traD	0	0	0	0	0	0	0	0	0	0	1
traN	0	0	0	0	0	0	0	0	0	0	1
tyeA	0	0	0	0	0	0	0	0	0	0	0
virB	0	0	0	0	0	0	0	0	0	1	0
virB1	0	0	0	0	0	0	0	0	0	0	0
virB4	0	0	0	0	0	0	0	0	0	0	0
xerC	0	0	0	0	0	0	0	0	0	1	0
xerD	0	0	0	0	0	0	0	0	0	0	1
yedJ	0	0	0	0	0	0	0	0	0	0	0
yegI	0	0	0	0	0	0	0	0	0	0	0
ygiW	0	0	0	0	0	0	0	0	0	0	0
yhcG	0	0	0	0	0	0	0	0	0	1	0
yiaC	0	0	0	0	0	0	0	0	0	0	0
yigB	0	0	0	0	0	0	0	0	0	1	0
yopB	0	0	0	0	0	0	0	0	0	0	0
yopN	0	0	0	0	0	0	0	0	0	0	0
yraJ	0	0	0	0	0	0	0	0	0	0	0
yscD	0	0	0	0	0	0	0	0	0	0	0
yscG	0	0	0	0	0	0	0	0	0	0	0
yscJ	0	0	0	0	0	0	0	0	0	0	0
yscN	0	0	0	0	0	0	0	0	0	0	0
yscU	0	0	0	0	0	0	0	0	0	0	0

Table S5. The number of mobile genetic elements (MGEs) identified in each *Photobacterium* genome. The symbiont species and the host fish family of origin are also indicated.

ID	Symbiont	Host	MGEs
LN-I.1	*P. leiognathi*	Leiognathidae	252
ljone.10.1	*P. leiognathi*	Leiognathidae	118
LR-VIII.1	*P. 'mandapamensis'*	Leiognathidae	94
LN-1a	*P. 'mandapamensis'*	Leiognathidae	55
ATCC 25521^{T}	*P. leiognathi*	Leiognathidae	52
lsplen.1.1	*P. 'mandapamensis'*	Leiognathidae	31
lnuch.19.1	*P. 'mandapamensis'*	Leiognathidae	18
ajapo.8.2	*P. 'mandapamensis'*	Acropomatidae	11
ajapo.8.1	*P. 'mandapamensis'*	Acropomatidae	11
lrivu.4.1	*P. leiognathi*	Leiognathidae	7
ajapo.3.1	*P. 'mandapamensis'*	Acropomatidae	6
calba.1.1	*P. kishitanii*	Other	3
LF-1a	*P. 'mandapamensis'*	Leiognathidae	2
Mot.1.1	*P. 'mandapamensis'*	Apogonidae	1
StJ.4.21	*P. 'mandapamensis'*	Apogonidae	1
StP.1.10	*P. 'mandapamensis'*	Apogonidae	1
svers.1.1	*P. 'mandapamensis'*	Apogonidae	1
lrivu.20.11	*P. 'mandapamensis'*	Leiognathidae	1
ajapo.4.1	*P. acropomis* sp. nov.	Acropomatidae	0
ajapo.5.5	*P. acropomis* sp. nov.	Acropomatidae	0
pjapo.1.1	*P. kishitanii*	Other	0
ckamo.1.1	*P. kishitanii*	Other	0
ahane.1.5	*P. kishitanii*	Acropomatidae	0
AJ-1a	*P. 'mandapamensis'*	Acropomatidae	0
ajapo.5.6	*P. 'mandapamensis'*	Acropomatidae	0
Mot.1.2	*P. 'mandapamensis'*	Apogonidae	0
Ik.8.2	*P. 'mandapamensis'*	Apogonidae	0
Kume.1.2	*P. 'mandapamensis'*	Apogonidae	0
SV.1.2	*P. 'mandapamensis'*	Apogonidae	0
Ik.8.1	*P. 'mandapamensis'*	Apogonidae	0
Kume.1.3	*P. 'mandapamensis'*	Apogonidae	0
StP.2.23	*P. 'mandapamensis'*	Apogonidae	0
SV.1.1	*P. 'mandapamensis'*	Apogonidae	0

*StJ.*4.81	*P. 'mandapamensis'*	Apogonidae	0
*SV.*5.1	*P. 'mandapamensis'*	Apogonidae	0
*Res.*4.1	*P. 'mandapamensis'*	Apogonidae	0
*lequu.*1.1	*P. 'mandapamensis'*	Leiognathidae	0

Table S6. List of unique genes found in *Photobacterium* strains *ajapo*.4.1 and *ajapo*.5.5 with available annotations as determined by the pangenome analysis with Roary (Page *et al.* 2015).

Gene ID	Predicted product
aat	lactate utilization protein C
aceK	bifunctional isocitrate dehydrogenase kinase/phosphatase
ahpD	Alkyl hydroperoxide reductase AhpD
ald	alanine dehydrogenase
amiC	Aliphatic amidase expression-regulating protein
amrA	AmmeMemoRadiSam system protein A
astB	N-acetyltransferase
betA	outer membrane beta-barrel protein
bfr	Bacterioferritin
birA	bifunctional biotin--[acetyl-CoA-carboxylase] ligase/biotin operon repressor BirA
bla	class A beta-lactamase
btuC	vitamin B12 ABC transporter ATP-binding protein BtuD
btuD	VanZ family protein
btuF	vitamin B12 ABC transporter permease BtuC
cadC	trans-2-enoyl-CoA reductase family protein
ccoS	cbb3-type cytochrome oxidase assembly protein CcoS
cobU	bifunctional adenosylcobinamide kinase/adenosylcobinamide-phosphate guanylyltransferase
dapH	2%2C3%2C4%2C5-tetrahydropyridine-2%2C6-dicarboxylate N-acetyltransferase
envC	MSHA biogenesis protein MshQ
flgA	flagellar basal body P-ring formation chaperone FlgA
flgK	flagellar hook-associated protein FlgK
flgN	flagellar export chaperone FlgN
gcvA	Transcriptional activator HlyU
gcvH	glycine cleavage system protein GcvH
group_13113	TraB/GumN family protein
group_13115	winged helix-turn-helix domain-containing protein
group_13116	alanyl-tRNA editing protein
group_13119	Transcriptional activator CadC
group_13121	DUF4868 domain-containing protein
group_13127	flagellar protein FlaG
group_13128	flagellar hook-length control protein FliK
group_13129	chemotaxis protein CheW
group_13134	pleiotropic regulatory protein RsmS
group_13136	DUF1496 domain-containing protein
group_13138	LysR family transcriptional regulator
group_13139	SDR family NAD(P)-dependent oxidoreductase
group_13140	substrate-binding domain-containing protein
group_13141	aldose 1-epimerase family protein
group_13142	DNA ligase

group_13145	L-ribulose-5-phosphate 3-epimerase, partial
group_13146	PTS ascorbate-specific subunit IIBC
group_13147	type II secretion system pilot lipoprotein GspS-beta
group_13149	methyltransferase
group_13150	lipocalin family protein
group_13153	SDR family oxidoreductase
group_13154	efflux RND transporter periplasmic adaptor subunit
group_13155	YcfL family protein
group_13156	phosphotransferase
group_13158	PilZ domain-containing protein
group_13160	EAL domain-containing protein
group_13161	DNA internalization-related competence protein ComEC/Rec2
group_13162	alkyl sulfatase dimerization domain-containing protein
group_13163	imelysin family protein
group_13167	MFS transporter
group_13171	DNA adenine methylase
group_13172	phosphorylase
group_13173	matE family protein
group_13174	alpha/beta fold hydrolase
group_13178	DeoR/GlpR family transcriptional regulator
group_13181	FUSC family protein
group_13182	high-potential iron-sulfur protein
group_13183	glycoside hydrolase family 18 protein
group_13184	J domain-containing protein
group_13185	glutathione peroxidase
group_13186	ABC transporter
group_13188	O-antigen ligase family protein
group_13189	rhodanese-like domain-containing protein
group_13191	pilin
group_13193	BON domain-containing protein
group_13194	YhdP family protein
group_13196	DUF2057 domain-containing protein
group_13199	porin
group_13203	SMR family transporter
group_13206	cell division protein ZapC
group_13210	DUF1318 domain-containing protein
group_13212	sulfite exporter TauE/SafE family protein
group_13216	SUMF1/EgtB/PvdO family nonheme iron enzyme
group_13222	LysE family translocator
group_13224	general secretion pathway protein GspB
group_13226	TAXI family TRAP transporter solute-binding subunit
group_13228	helix-turn-helix domain-containing protein
group_13229	HesA/MoeB/ThiF family protein

group_13230	DoxX family protein
group_13231	AsmA family protein
group_13233	type II secretion system protein N
group_13234	ATPase
group_13240	beta-ketoacyl synthase chain length factor
group_13241	AMP-binding protein
group_13243	AraC family transcriptional regulator
group_13245	PhzF family phenazine biosynthesis protein
group_13246	polysaccharide deacetylase family protein
group_13247	glycosyltransferase
group_13252	dTDP-4-dehydrorhamnose 3,5-epimerase
group_13253	tRNA lysidine(34) synthetase TilS
group_13260	DNA-processing protein DprA
group_13261	methyl-accepting chemotaxis protein
group_13264	gamma carbonic anhydrase family protein
group_13265	N-6 DNA methylase
group_13266	ParB/RepB/Spo0J family partition protein
group_13267	heme ABC transporter ATP-binding protein
group_13268	iron ABC transporter permease
group_13269	ABC transporter substrate-binding protein
group_13270	Zn(2+)-responsive transcriptional regulator
group_13271	AlpA family transcriptional regulator
group_13272	D-2-hydroxyacid dehydrogenase
group_13273	LruC domain-containing protein
group_13274	plasmid replication initiator TrfA
group_13276	helicase-related protein
group_13278	L%2CD-transpeptidase family protein
group_13279	YjjW family glycine radical enzyme activase
group_13280	McrC family protein
group_13282	VirK/YbjX family protein
group_13283	DUF6236 family protein
group_13284	Y-family DNA polymerase
group_13285	FRG domain-containing protein
group_13289	TolC family protein
group_13291	lytic transglycosylase F
group_13295	extracellular solute-binding protein
group_13296	C40 family peptidase
group_13301	mechanosensitive ion channel
group_13302	putative adenosine monophosphate-protein transferase Fic
group_13305	YggN family protein
group_13306	phosphatase PAP2 family protein
group_13309	gamma-glutamylcyclotransferase family protein
group_13310	biotin-dependent carboxyltransferase family protein

group_13312	5-oxoprolinase subunit PxpA
group_13313	PfkB family carbohydrate kinase
group_13315	3'-5' exonuclease
group_13316	type I-F CRISPR-associated protein Csy3
group_13317	glutaredoxin domain-containing protein
group_13319	helix-turn-helix transcriptional regulator
group_13322	NUDIX domain-containing protein
group_13324	HNH endonuclease signature motif containing protein
group_13327	Lon protease
group_13330	leucyl/phenylalanyl-tRNA--protein transferase
group_13333	siderophore-interacting protein
group_13335	fimbrial protein
group_13338	uroporphyrinogen-III C-methyltransferase
group_13339	LON peptidase substrate-binding domain-containing protein
group_13340	lipase
group_13341	siderophore ferric iron reductase
group_13344	glyceraldehyde 3-phosphate dehydrogenase N-terminal domain-containing protein
group_13350	amino acid ABC transporter ATP-binding protein
group_13356	glucosaminidase domain-containing protein
group_13357	aromatic amino acid transport family protein
group_13358	undecaprenyl-phosphate glucose phosphotransferase
group_13361	bi-domain-containing oxidoreductase
group_13364	phosphomannomutase
group_13366	UDP-N-acetyl-D-mannosamine dehydrogenase
group_13368	mannose-1-phosphate guanylyltransferase/mannose-6-phosphate isomerase
group_13369	glutathione S-transferase family protein
group_13370	glycosyltransferase family 4 protein
group_13373	D-hexose-6-phosphate mutarotase
group_13374	nucleoside-diphosphate sugar epimerase/dehydratase
group_13375	(deoxy)nucleoside triphosphate pyrophosphohydrolase
group_13376	SEC-C metal-binding domain-containing protein
group_13377	NAD(P)H-binding protein
group_13382	ComEA family DNA-binding protein
group_13383	response regulator
group_13388	Undecaprenyl-phosphate alpha-N-acetylglucosaminyl 1-phosphate transferase
group_13394	outer membrane protein assembly factor BamE
group_13397	NAD-dependent epimerase
group_13399	YfcL family protein
group_13400	PTS sugar transporter subunit IIA
group_13402	acetate kinase
group_13404	NO-inducible flavohemoprotein
group_13407	Oxygen-dependent choline dehydrogenase
group_13411	2-oxoglutarate translocator

group_13412	OmpA family protein
group_13413	sugar transferase
group_13415	sugar transporter
group_13419	oligosaccharide flippase family protein
group_13425	M36 family metallopeptidase
group_13431	copper-binding protein
group_13432	HAAAP family serine/threonine permease
group_13438	sugar O-acetyltransferase
group_13441	NAD(+)--arginine ADP-ribosyltransferase Chelt
group_13444	SPOR domain-containing protein
group_13446	XRE family transcriptional regulator
group_13447	formate dehydrogenase subunit gamma
group_13448	M20/M25/M40 family metallo-hydrolase
group_13449	CPBP family intramembrane metalloprotease
group_13450	cytochrome c
group_13451	dihydrolipoamide acetyltransferase family protein
group_13454	CoA pyrophosphatase
group_13457	ribosome biogenesis GTPase YlqF
group_13460	retention module-containing protein
group_13463	S9 family peptidase
group_13464	TOBE domain-containing protein
group_13465	DUF3703 domain-containing protein
group_13467	metal ABC transporter permease
group_13469	maleylacetoacetate isomerase
group_13470	HAD family phosphatase
group_13471	hybrid sensor histidine kinase/response regulator
group_13472	phosphoethanolamine--lipid A transferase
group_13473	aminoglycoside phosphotransferase family protein
group_13474	DMT family transporter
group_13475	nucleotidyltransferase family protein
group_13477	histidine phosphatase family protein
group_13478	cobyric acid synthase
group_13480	NfeD family protein
group_13482	GIY-YIG nuclease family protein
group_13483	YdcF family protein
group_13484	delta-aminolevulinic acid dehydratase
group_13485	ABC transporter permease
group_13486	protein-methionine-sulfoxide reductase heme-binding subunit MsrQ
group_13488	SCO family protein
group_13490	chemotaxis protein CheV
group_13492	MDR family MFS transporter
group_13493	DmsC/YnfH family molybdoenzyme membrane anchor subunit
group_13494	isochorismatase family protein

group_13495	toxin-antitoxin system YwqK family antitoxin
group_13496	DUF5011 domain-containing protein
group_13498	siroheme synthase
group_13499	serine protease
group_13501	TIGR02450 family Trp-rich protein
group_13504	ADP-L-glycero-D-manno-heptose-6-epimerase
group_13505	carbamoyl-phosphate synthase large subunit
group_13509	sensor histidine kinase VxrA
group_13511	nicotinate-nicotinamide nucleotide adenylyltransferase
group_13512	AraC family ligand binding domain-containing protein
group_13517	DDE-type integrase/transposase/recombinase
group_13519	Nramp family divalent metal transporter
group_13520	C-terminal helicase domain-containing protein, partial
group_13522	AAA domain-containing protein
group_13523	NERD domain-containing protein
group_13524	DinI-like family protein
group_13526	Gfo/Idh/MocA family oxidoreductase
group_13527	TniQ family protein
group_13528	class I SAM-dependent DNA methyltransferase
group_13529	signal peptidase I
group_13533	excalibur calcium-binding domain-containing protein
group_13534	DUF4347 domain-containing protein
group_13536	DUF11 domain-containing protein
group_13540	NlpC/P60 family protein
group_13541	choloylglycine hydrolase family protein
group_13548	YdbL family protein
group_13552	right-handed parallel beta-helix repeat-containing protein
group_13553	ParA family protein
group_13556	reverse transcriptase N-terminal domain-containing protein
group_13562	enoyl-CoA hydratase-related protein
group_13563	hydroxymethylglutaryl-CoA lyase
group_13565	D-alanyl-D-alanine carboxypeptidase family protein
group_13570	nuclear transport factor 2 family protein
group_13572	type IV conjugative transfer system coupling protein TraD
group_13578	RepB family plasmid replication initiator protein
group_13579	YadA-like family protein
group_13580	DNA cytosine methyltransferase
group_13587	thioredoxin fold domain-containing protein
group_13591	SH3 domain-containing protein
group_13595	glycosyl hydrolase family 18 protein
group_13596	pyrimidine 5'-nucleotidase
group_13598	N-succinylarginine dihydrolase
group_13600	choice-of-anchor I family protein

group_13602	type II secretion system F family protein
group_13605	cryptochrome/photolyase family protein
group_13606	DASH family cryptochrome
group_13607	prepilin-type N-terminal cleavage/methylation domain-containing protein
group_13616	rod-binding protein
group_13618	SslE/AcfD family lipoprotein zinc metalloprotease
group_13622	cytochrome b
group_13624	HlyD family secretion protein
group_13625	DUF2895 family protein
group_13626	HAD-IA family hydrolase
group_13628	TIGR03752 family integrating conjugative element protein
group_13630	conjugative transfer ATPase
group_13632	phospholipase A
group_13633	LuxR family transcriptional regulator
group_13634	Type III restriction-modification system restriction subunit
group_13635	type III restriction-modification system endonuclease
group_13637	BatD family protein
group_13638	site-specific DNA-methyltransferase
group_13641	glutamine-hydrolyzing carbamoyl-phosphate synthase small subunit
group_13649	LysR substrate-binding domain-containing protein
group_13650	diguanylate cyclase domain protein
group_13652	ABC transporter ATP-binding protein
group_13657	endonuclease/exonuclease/phosphatase family protein
group_13658	SRPBCC family protein
group_13659	L-2-hydroxyglutarate oxidase
group_13660	lateral flagellin LafA
group_13661	GNAT family N-acyltransferase
group_13662	YebC/PmpR family DNA-binding transcriptional regulator
group_13665	peptidoglycan DD-metalloendopeptidase family protein
group_13666	GNAT family N-acetyltransferase
group_13670	phosphoethanolamine transferase
group_13674	VRR-NUC domain-containing protein
group_13675	OsmC family protein
group_13680	lytic transglycosylase domain-containing protein
group_13681	TrbC/VirB2 family protein
group_13683	Ivy family c-type lysozyme inhibitor
group_13685	heme NO-binding domain-containing protein
group_13687	penicillin-binding protein 2
group_13689	DUF2955 domain-containing protein
group_13691	DEAD/DEAH box helicase
group_13696	RimK/LysX family protein
group_13701	LuxG
group_13702	HAD family hydrolase

group_13703	acetoacetate--CoA ligase
group_13704	septal ring lytic transglycosylase RlpA family protein
group_13706	replication initiation protein
group_13709	DUF2786 domain-containing protein
group_13710	Na(+)-translocating NADH-quinone reductase subunit B
group_13711	NADH:ubiquinone reductase (Na(+)-transporting) subunit B
group_13712	bifunctional molybdopterin-guanine dinucleotide biosynthesis adaptor protein MobB/molybdopterin molybdotransferase MoeA
group_13713	MarR family winged helix-turn-helix transcriptional regulator
group_13717	spermidine/putrescine ABC transporter substrate-binding protein
group_13721	nuclease-related domain-containing protein
group_13722	transcriptional antiterminator
group_13724	aspartoacylase
group_13728	CHAD domain-containing protein
group_13731	1-acylglycerol-3-phosphate O-acyltransferase
group_13733	RNA-directed DNA polymerase
group_13735	YihD family protein
group_13736	DM13 domain-containing protein
group_13739	RutC family protein YjgH
group_13740	DJ-1/PfpI family protein
group_13741	rod shape-determining protein
group_13750	YfiR family protein
group_13753	fluoride efflux transporter CrcB
group_13754	questin oxidase family protein
group_13760	Putative NAD(+)--arginine ADP-ribosyltransferase Vis
group_13761	restriction endonuclease subunit S
group_13762	MATE family efflux transporter
group_13763	leukocidin family pore-forming toxin
group_13766	type B 50S ribosomal protein L31
group_13767	pseudouridine synthase
group_13768	transcriptional regulator GcvA
group_13770	cupin domain-containing protein
group_13771	FAD-dependent oxidoreductase
group_13772	SEL1-like repeat protein
group_13773	hydrogenase maturation nickel metallochaperone HypA
group_13774	carbamoyltransferase HypF
group_13779	response regulator transcription factor
group_13780	histidine kinase dimerization/phospho-acceptor domain-containing protein
group_13781	MerR family transcriptional regulator
group_13783	biopolymer transporter ExbD
group_13785	Qnr family pentapeptide repeat protein
group_13786	shikimate kinase
group_13788	amine oxidase; protein
group_13790	selenocysteine-specific translation elongation factor

group_13791	RidA family protein
group_13792	ATP-binding cassette domain-containing protein
group_13793	malonyl-ACP O-methyltransferase BioC
group_13795	hybrid-cluster NAD(P)-dependent oxidoreductase
group_13798	copper chaperone PCu(A)C
group_13799	phytanoyl-CoA dioxygenase family protein
group_13802	lactate dehydrogenase
group_13803	isoprenylcysteine carboxylmethyltransferase family protein
group_13806	VOC family protein
group_13808	asparagine synthase-related protein
group_13809	methylated-DNA--[protein]-cysteine S-methyltransferase
group_13811	site-specific integrase
group_13814	murein L%2CD-transpeptidase catalytic domain family protein
group_13816	ATP-binding protein
group_13818	PTS transporter subunit EIIC
group_13821	HEPN domain-containing protein
group_13825	phosphate regulon sensor histidine kinase PhoR
group_13830	SIS domain-containing protein
group_13831	zincin-like metallopeptidase domain-containing protein
group_13832	ParB family protein
group_13833	TonB-dependent receptor
group_13834	BamA/TamA family outer membrane protein
group_13837	Integrase core domain protein
group_13839	UvrABC system protein B
group_13840	DNA sulfur modification protein DndB
group_13841	nitrous oxide-stimulated promoter family protein
group_13844	DUF2264 domain-containing protein
group_13852	conjugal transfer protein TraG N-terminal domain-containing protein
group_13853	acyltransferase family protein
group_13855	membrane protein
group_13856	recombinase RecT
group_13858	GGDEF domain-containing protein
group_13861	spondin domain-containing protein
group_13862	transposase
group_13865	phosphoadenosine phosphosulfate reductase family protein
group_13868	DNA topoisomerase
group_13870	pilus assembly protein PilP
group_13871	bifunctional aspartate kinase/homoserine dehydrogenase II
group_13873	Spy/CpxP family protein refolding chaperone
group_13874	murein hydrolase activator EnvC
group_13875	TetR/AcrR family transcriptional regulator
group_13879	glycosyltransferase family 25 protein
group_13881	lysoplasmalogenase

group_13882	DUF488 domain-containing protein
group_13883	patatin family protein
group_13887	YqaJ viral recombinase family protein
group_13889	DUF3150 domain-containing protein
group_13891	VWA domain-containing protein
group_13893	Prophage integrase IntA
group_13894	toprim domain-containing protein
group_13895	recombinase family protein
group_13900	M23 family metallopeptidase
group_13901	TatD family hydrolase
group_13903	DUF6035 family protein
group_13904	putative 4-hydroxy-4-methyl-2-oxoglutarate aldolase
group_13905	vitamin B12 ABC transporter substrate-binding protein BtuF
group_16257	iron-containing alcohol dehydrogenase
group_252	IS30 family transposase ISSde3
group_3915	GEVED domain-containing protein
group_504	RNA polymerase sigma factor SigZ
group_5951	AAA family ATPase
group_7226	retention module-containing protein
group_7348	GEVED domain-containing protein
group_755	L-type lectin domain-containing protein; hypothetical protein
gspC	type II secretion system protein GspC
gspS2	trypsin-like serine protease
hmpA	NnrS family protein
hppD	4-hydroxyphenylpyruvate dioxygenase
hypB	hydrogenase nickel incorporation protein HypB
intA	potassium channel family protein
kdsA	3-deoxy-8-phosphooctulonate synthase
kdsB	3-deoxy-manno-octulosonate cytidylyltransferase
lepB	sigma-70 family RNA polymerase sigma factor
lhgO	KpsF/GutQ family sugar-phosphate isomerase
lptB	LPS export ABC transporter ATP-binding protein
maiA	M66 family metalloprotease
mddA	Methanethiol S-methyltransferase
menC	o-succinylbenzoate synthase
menD	2-succinyl-5-enolpyruvyl-6-hydroxy-3- cyclohexene-1-carboxylic-acid synthase
menH	2-succinyl-6-hydroxy-2%2C 4-cyclohexadiene-1-carboxylate synthase
mnmC	bifunctional tRNA (5-methylaminomethyl-2-thiouridine)(34)-methyltransferase MnmD/FAD-dependent 5-carboxymethylaminomethyl-2- [illegible]
mrdA	patatin-like phospholipase family protein
mshA	D-inositol-3-phosphate glycosyltransferase
msrA	peptide-methionine (S)-S-oxide reductase MsrA
msrB	PepSY domain-containing protein
msrB	peptide-methionine (R)-S-oxide reductase MsrB

msrQ	protein disulfide oxidoreductase
murB	UDP-N-acetylglucosamine 2-epimerase (non-hydrolyzing)
murE	UDP-N-acetylmuramate dehydrogenase
mutL	DNA mismatch repair endonuclease MutL
nudF	ADP-ribose diphosphatase
phrB	deoxyribodipyrimidine photo-lyase
putA	bifunctional proline dehydrogenase/L-glutamate gamma-semialdehyde dehydrogenase PutA
pxpB	5-oxoprolinase subunit PxpB
rfbC	dTDP-4-dehydrorhamnose 3%2C5-epimerase
rffH	Glucose-1-phosphate thymidylyltransferase 2
rpoD	RNA polymerase sigma factor RpoD
rpoS	RNA polymerase sigma factor RpoS
rsmS	pirin family protein
sfaA	S-fimbrial protein subunit SfaA
sigZ	RNA polymerase sigma factor RpoS
spnR	dTDP-4-dehydro-2%2C3%2C6-trideoxy-D-glucose 4-aminotransferase
thiD	bifunctional hydroxymethylpyrimidine kinase/phosphomethylpyrimidine kinase
tilS	transporter substrate-binding domain-containing protein
tpx	Thiol peroxidase
ureB	Urease subunit beta
ureC	Urease subunit alpha
ureD	Urease accessory protein UreD
ureE	Urease accessory protein UreE
ureF	Urease accessory protein UreF
uvrB	Urease subunit gamma
vxrA	sensor domain-containing diguanylate cyclase
wecA	UDP-N-acetylmuramoyl-L-alanyl-D-glutamate--2%2C 6-diaminopimelate ligase
wecB	UDP-N-acetylglucosamine 2-epimerase (non-hydrolyzing)
wecC	tyrosine-type recombinase/integrase
yjgH	ROK family protein
yjjG	putative protein YqjZ
ylqF	rhomboid family intramembrane serine protease
ypdF	Aminopeptidase YpdF
yvbK	putative N-acetyltransferase YvbK
zntR	zinc ribbon domain-containing protein

TableS7. List of genes present in *P. 'mandapamensis'* strains but absent in *P. leiognathi* as identified by Roary.

Gene ID	Function
ald	Alanine dehydrogenase
ccoP2	Cbb3-type cytochrome c oxidase subunit CcoP2
cmpB	Bicarbonate transport system permease protein CmpB
cysG	Siroheme synthase
dgcM	diguanylate cyclase
epsC	Type II secretion system protein C
epsD	Secretin GspD
epsL	Type II secretion system protein L
epsM	Type II secretion system protein M
xcpW	Type II secretion system protein J
gspK	Putative type II secretion system protein K
glmS	Glutamine--fructose-6-phosphate aminotransferase [isomerizing]
gph	Phosphoglycolate phosphatase
group_1896	SDR family oxidoreductase
group_1998	hypothetical protein
group_2216	DUF11 domain-containing protein
group_2217	selenocysteine-specific translation elongation factor
group_2653	ABC transporter permease subunit
group_3505	Wzz/FepE/Etk N-terminal domain-containing protein
group_3506	hypothetical protein
group_4431	3-phosphoserine/phosphohydroxythreonine transaminase
group_4800	L,D-transpeptidase family protein
group_4904	hypothetical protein
group_5960	MFS transporter
group_7070	hypothetical protein
group_7122	hypothetical protein
group_7381	hypothetical protein
group_8398	porin
group_8399	DUF6602 domain-containing protein
lplA	Lipoate-protein ligase A
nasD	Nitrite reductase [NAD(P)H]
nirD	Nitrite reductase (NADH) small subunit
nrtD	Nitrate import ATP-binding protein NrtD
petA	Ubiquinol-cytochrome c reductase iron-sulfur subunit

rodZ	Cytoskeleton protein RodZ
sdaC	Serine transporter
typA	GTP-binding protein TypA/BipA
ybhF	putative multidrug ABC transporter ATP-binding protein YbhF
ycbB	putative L,D-transpeptidase YcbB
yohC	Inner membrane protein YohC

TableS8. List of genes present in *P. leiognathi* strains but absent in *P. 'mandapamensis'* as identified by Roary.

Gene ID	Function
group_11807	Type II secretion system protein M
group_11808	Putative type II secretion system protein K
group_11809	Type II secretion system protein J
group_11810	Secretin GspD
group_11811	Type II secretion system protein C
group_15629	hypothetical protein
group_16406	Ubiquinol-cytochrome c reductase iron-sulfur subunit
group_4351	Alanine dehydrogenase
group_7972	Type II secretion system protein L
group_7996	carboxymuconolactone decarboxylase
group_7998	Serine transporter
napA	Periplasmic nitrate reductase
ndhC (*nuoA*)	NAD(P)H-quinone oxidoreductase subunit 3
nuoB	NADH-quinone oxidoreductase subunit B
nuoC	NADH-quinone oxidoreductase subunit C/D
nuoF	NADH-quinone oxidoreductase subunit F
nuoH	NADH-quinone oxidoreductase subunit H
nuoI	NADH-quinone oxidoreductase subunit I
nuoJ	NADH-quinone oxidoreductase subunit J
nuoK	NADH-quinone oxidoreductase subunit K
nuoL	NADH-quinone oxidoreductase subunit L
nuoM	NADH-quinone oxidoreductase subunit M
nuoN	NAD(P)H-quinone oxidoreductase subunit 2, chloroplastic
typA	GTP-binding protein TypA/BipA
